# Migratory culture, population structure and stock identity in North Pacific beluga whales *(Delphinapterus leucas)*

**DOI:** 10.1371/journal.pone.0194201

**Published:** 2018-03-22

**Authors:** Greg O’Corry-Crowe, Robert Suydam, Lori Quakenbush, Brooke Potgieter, Lois Harwood, Dennis Litovka, Tatiana Ferrer, John Citta, Vladimir Burkanov, Kathy Frost, Barbara Mahoney

**Affiliations:** 1 Harbor Branch Oceanographic Institute, Florida Atlantic University, Fort Pierce, Florida, United States of America; 2 North Slope Borough Department of Wildlife Management, Barrow, Alaska, United States of America; 3 Alaska Department of Fish and Game, Fairbanks, Alaska, United States of America; 4 Fisheries and Oceans Canada, Yellowknife, Northwest Territories, Canada; 5 Marine Mammal Laboratory, ChukotTINRO, Anadyr, Chukotka, Russia; 6 North Pacific Wildlife Consulting, Marine Mammal Laboratory, Seattle, Washington, United States of America; 7 University of Alaska, School of Fisheries and Ocean Science, Kailua Kona, Hawaii, United States of America; 8 National Marine Fisheries Service, Anchorage, Alaska, United States of America; Sanya Institute of Deep-sea Science and Engineering Chinese Academy of Sciences, CHINA

## Abstract

The annual return of beluga whales, *Delphinapterus leucas*, to traditional seasonal locations across the Arctic may involve migratory culture, while the convergence of discrete summering aggregations on common wintering grounds may facilitate outbreeding. Natal philopatry and cultural inheritance, however, has been difficult to assess as earlier studies were of too short a duration, while genetic analyses of breeding patterns, especially across the beluga’s Pacific range, have been hampered by inadequate sampling and sparse information on wintering areas. Using a much expanded sample and genetic marker set comprising 1,647 whales, spanning more than two decades and encompassing all major coastal summering aggregations in the Pacific Ocean, we found evolutionary-level divergence among three geographic regions: the Gulf of Alaska, the Bering-Chukchi-Beaufort Seas, and the Sea of Okhotsk (*Φ*_st_ = 0.11–0.32, *R*_st_ = 0.09–0.13), and likely demographic independence of (*F*_st-mtDNA_ = 0.02–0.66), and in many cases limited gene flow (*F*_st-nDNA_ = 0.0–0.02; *K* = 5–6) among, summering groups within regions. Assignment tests identified few immigrants within summering aggregations, linked migrating groups to specific summering areas, and found that some migratory corridors comprise whales from multiple subpopulations (P_BAYES_ = 0.31:0.69). Further, dispersal is male-biased and substantial numbers of closely related whales congregate together at coastal summering areas. Stable patterns of heterogeneity between areas and consistently high proportions (~20%) of close kin (including parent-offspring) sampled up to 20 years apart within areas (*G* = 0.2–2.9, *p*>0.5) is the first direct evidence of natal philopatry to migration destinations in belugas. Using recent satellite telemetry findings on belugas we found that the spatial proximity of winter ranges has a greater influence on the degree of both individual and genetic exchange than summer ranges (r_winter_-*F*_st-mtDNA_ = 0.9, r_summer_-*F*_st-nDNA_ = 0.1). These findings indicate widespread natal philopatry to summering aggregation and entire migratory circuits, and provide compelling evidence that migratory culture and kinship helps maintain demographically discrete beluga stocks that can overlap in time and space.

## Introduction

In migratory species social learning, seasonal movements and the use of geographically separate habitats during the annual cycle can foster the cultural inheritance of migration routes and complex patterns of dispersal, gene flow and population structure [1, 2, 3]. These, in turn, have implications for gene-culture coevolution [4] and create novel challenges for management and conservation, including the identification of management units, the assignment of migrating animals to population of origin, and the assessment of risk at the population level.

The primary factors influencing population subdivision in migratory species include; (1) Non-uniform patterns of seasonal resource distribution which can foster philopatry to geographically discrete migration destinations (e.g., feeding or breeding grounds) that can promote population divergence over time [2, 3]. (2) Such population units, however, may overlap during migration or co-occur at another migration destination or staging area for part of the year, facilitating dispersal and interbreeding. (3) In species with extended periods of parental care natal philopatry and thus population divergence may involve parentally directed learning of migration routes termed migratory culture [1, 5–7]. (4) Structuring in migratory species also depends on which sex disperses [8, 9] with sex-biased dispersal potentially leading to the demographic isolation of population units even in the face of gene flow and seasonal overlap [10]. (5) Patterns of dispersal and heterogeneity in migratory species can be shaped by geography and other environmental factors (e.g., water temperature, weather patterns, sea-ice cover) that directly dictate the path and timing of migration, for example [11], and thus the level and duration of population overlap. And finally (6), glacial history, especially at higher latitudes, where the historical location of refugial populations and the sequence in which new habitat emerged following deglaciation can have a lasting effect on contemporary patterns of distribution and migration, and thus dispersal and population structure [12].Disentangling the contributions of such factors to migration, dispersal and structuring in migratory species is challenging. Genetic investigations offer huge potential in resolving the interplay between behaviors, the environment, and demographic history in shaping seasonal movements, dispersal and population structure in these species.

The beluga whale, *Delphinapterus leucas*, is a mid-sized cetacean of Arctic and sub-Arctic waters where all these factors likely interplay to shape contemporary migration, dispersal, breeding, and population structure patterns. In this study we use spatial and temporal patterns of genetic variation and individual relatedness to test a series of hypotheses about these aspects of belugas in the Pacific.

Across much of their range belugas migrate between wintering areas at or near the southern margin of the sea-ice and summering grounds in seasonally more open water farther north where they feed, molt and raise their young [13, 14]. Many Arctic populations are migratory with some completing annual circuits in excess of 6,000km while subarctic populations are less so, often exhibiting substantial overlap between winter and summer ranges [14–19]. Highly gregarious, beluga whales congregate by the thousands at several geographically discrete nearshore locations following ice-breakup in summer [20, 21]. Breeding is believed to occur primarily in winter and early spring [22], and adult males may select different habitats than females and younger animals at certain times of year [16, 23–25]. These attributes, combined with the potential for intersecting migration routes [26] and for separate summering groups sharing the same wintering ground during the period of peak mating [27], contribute to complex patterns of dispersal and population structure in beluga whales, raise the possibility of stable migratory cultures, and create unique challenges for species management, including the identification and monitoring of stocks.

**Fig 1:**
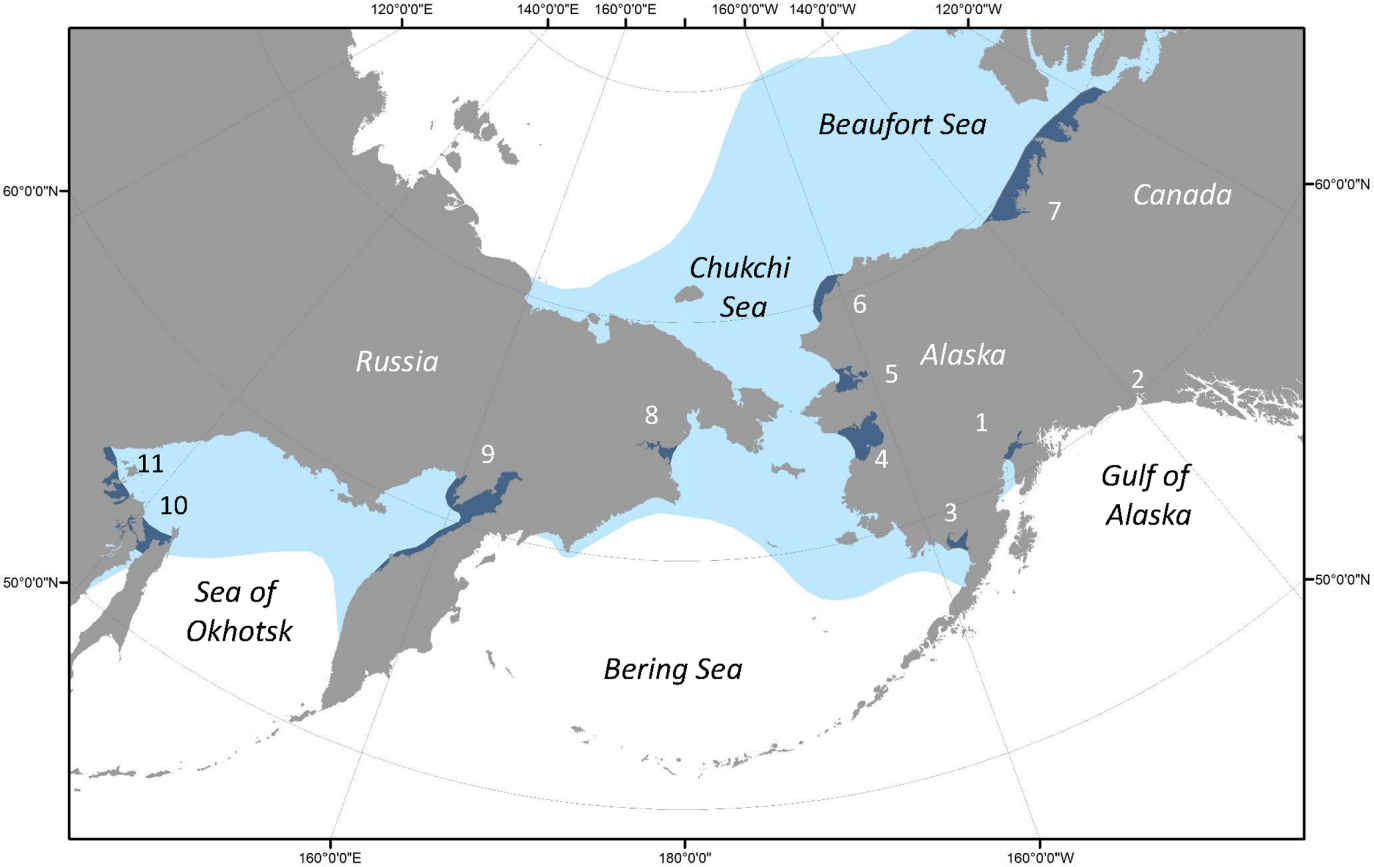
Distribution (light blue) of beluga whales, *Delphinapterus leucas*, in the North Pacific Ocean. The ten major nearshore concentration areas during the summer months are highlighted (dark blue). These areas along with a small resident group of beluga whales in the Gulf of Alaska are numbered according to Table 1.

**Fig 2:**
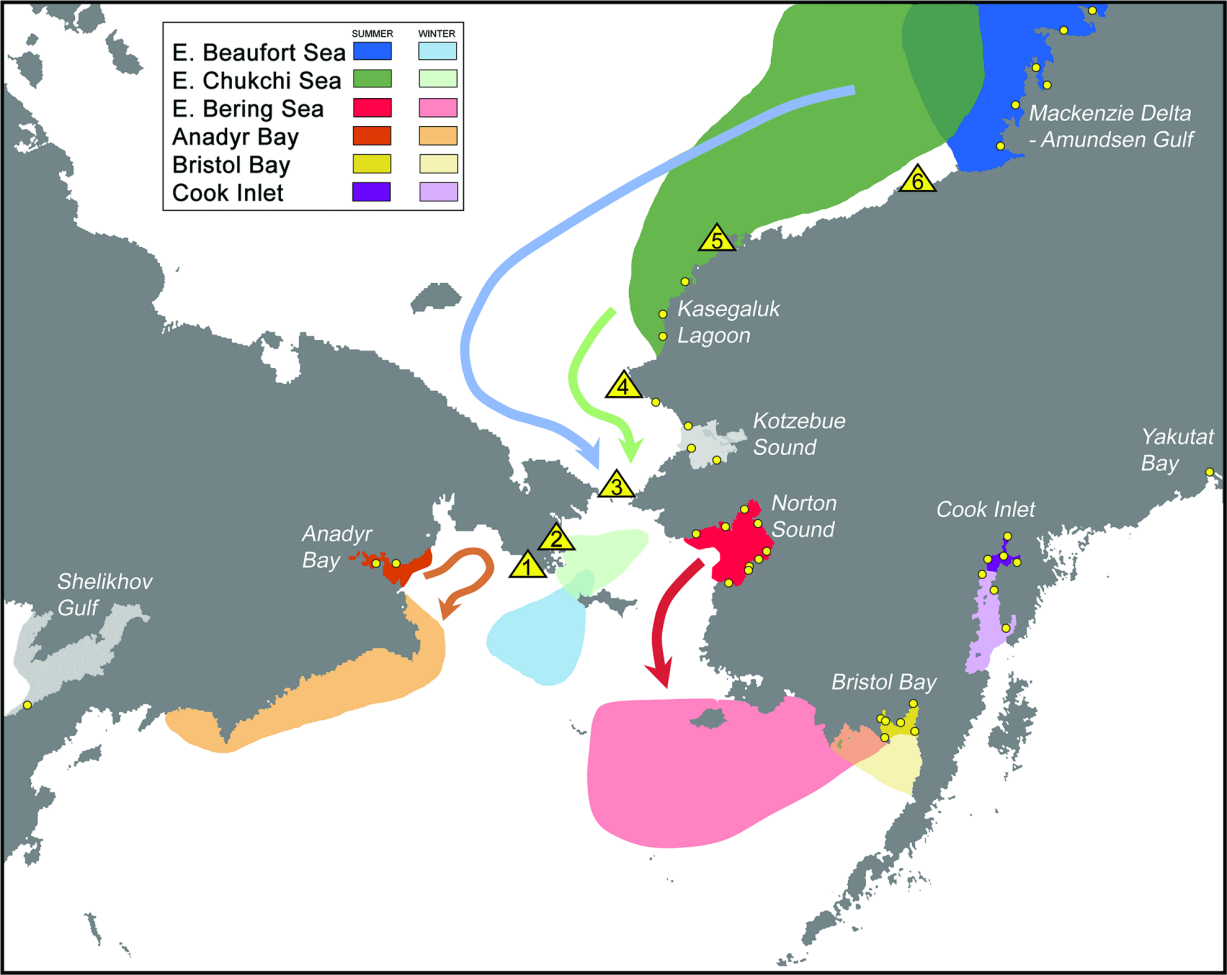
Migration routes and sampling locations of beluga whales from the major summer concentration areas in the Bering, Chukchi and Beaufort Seas and from the Gulf of Alaska. Summering and wintering areas, and migration routes were inferred from a combination of satellite telemetry, aerial and shore based sightings, and Traditional Ecological Knowledge (TEK). Sampling sites are indicated by yellow circles and in the case of migration by triangles.

In the Pacific, beluga whales inhabit three geographically distinct regions; (1) the Gulf of Alaska, (2) the Sea of Okhotsk, and (3) the Bering, Chukchi and Beaufort Seas, termed the BCB region (Fig 1). Within the BCB region, there are six major summer aggregation areas near shore that are labeled in Fig 1 as follows: Bristol Bay (^#^3) and Norton Sound (^#^4) in the eastern Bering Sea, Kotzebue Sound (^#^5) and off Kasegaluk Lagoon (^#^6) in the eastern Chukchi Sea, Mackenzie Delta-Amundsen Gulf region (^#^7) of the eastern Beaufort Sea, and Anadyr Bay (^#^8) in the western Bering Sea (Figs 1 and 2). There are also three discrete summering locations in the Okhotsk Sea (^#^9, ^#^10, and ^#^11), and a small resident population in Cook Inlet (^#^1) and isolated group in Yakutat Bay (^#^2) in the Gulf of Alaska (Fig 1).

The available evidence indicates that beluga whales in the BCB region overwinter in the northern and central Bering Sea at or near the southern extent of the sea ice [18, 28–31] (Fig 2). The hour-glass shape of the Bering-Chukchi basin constricts the migration of Chukchi and Beaufort summering groups through the narrow (85km wide) Bering Strait en route to and from wintering areas in the Bering Sea (Fig 2). This geographic feature, in concert with the timing and pattern of ice breakup and formation, likely exerts a substantial influence on when whales migrate, what routes they use, where they overwinter and to what extent separate summering groups overlap in winter. A recent study summarizing satellite telemetry data from five of the six BCB summering groups (i.e., excluding Kotzebue Sound) revealed that whales from discrete summering groups may also have traditional wintering areas with varying degrees of overlap [31]. Considering the peak breeding season for beluga whales is during winter and early spring [22], there is potential for extensive genetic and individual exchange among BCB summering groups both on migration and during the winter.

A series of genetic studies have been conducted on beluga whales in the western Nearctic (Alaska and northwestern Canada) and in the eastern Palearctic (Russian far east). A number revealed substantial mtDNA differentiation among geographically isolated populations and among some summering groups that likely reflects long-established patterns of female-mediated philopatry and demographic isolation [32–36] leading to their identification as demographically distinct management stocks [37, 38]. A few studies have documented lower levels of nDNA (microsatellite) heterogeneity among geographic strata compared to mtDNA which has been taken as evidence of extensive male-mediated gene flow among summering groups, possibly on shared wintering grounds [27, 39].

These earlier studies, however, have a number of limitations with respect to resolving patterns of migration, dispersal and gene flow of Pacific beluga whales basin-wide. All were of too short a duration to directly test for natal philopatry and migratory culture in this long-lived mammal, and to assess the stability of migration and dispersal patterns over ecological time frames. All had a regional focus that risked incomplete assessments of the molecular and behavioral ecology of this highly vagile species in an environment with few geographic barriers. The failure to include all populations, for example, can lead to inaccurate cluster analyses and erroneous assignment tests. Low marker number and sample size in many cases limited interpretations of observed patterns of differentiation. Furthermore, differing laboratory conditions prevented the comparison of nDNA (microsatellite) data sets among studies and populations, while few whales were sampled on migration or outside traditional coastal summering areas.

In this study, we investigate migration behavior, dispersal, population structure and stock identity in Pacific beluga whales. We include whales from all major coastal concentration areas in the north Pacific for the first time. We analyze 1,444 samples for both mtDNA and eight microsatellite loci. We conduct analyses on a further 203 Russian Far East whales from the literature [39] for a total of 1,647 whales from across their Pacific range. We test hypotheses on how the spatial proximity of summering and wintering areas may influence levels of dispersal and interbreeding among discrete summering aggregations, some of the findings of which have recently been referred to in the tracking paper by Citta et al. [31]. We include previously unsampled and undersampled locations, greatly expand the analysis of migrating whales, and extend the sampling time frame in many areas to encompass more than three decades from 1978–2010. With this extended time frame we assess patterns of kinship within groups and among years, determine the stability of migration patterns and structure over ecological time frames, and further investigate the population origins of beluga whales in one area (Kotzebue Sound, Alaska) that has witnessed dramatic changes in the pattern of annual return of beluga whales in summer. Finally, we explore how model-based cluster analysis of population genetic structure and Bayesian inference of recent rates of dispersal may be influenced by sample size, uneven sample numbers, locus number and differing degrees of subdivision.

## Materials and methods

### Sample collection and laboratory analysis

All samples were collected under the authority of U.S. Marine Mammal Protection Act permits issued by the National Marine Fisheries Service, Russian Marine Mammal permits, and the Dept. of Fisheries and Oceans, Canada scientific licenses. Tissue samples were collected from 1,444 beluga whales at 45 locations across 15 geographic strata in in the Gulf of Alaska, the Bering, Chukchi and Beaufort Seas, and the Sea of Okhotsk between 1978–2010 (Table 1, Fig 1). A more detailed summary of sample numbers across the four distinct sampling periods of this study are provided in S1 Appendix. Tissues were preserved in 20% dimethyl sulfoxide (DMSO) saturated with NaCl. Total DNA was extracted from each sample using standard cell lysis-high-salt extraction protocols, and each sample was screened for variation within 410bp of the mtDNA control region according to previously published methods [32, 33]. Total DNA was also extracted from a number of dried tissue and teeth samples using standard silica-based ancient DNA methods [40, 41]. The gender of each sample was determined by PCR-based methods [42] and all samples were analyzed for variation within eight microsatellite loci (S1 Table) typed on beluga whales [43] or other cetacean species [44, 45]. Earlier studies including an analysis of 288 belugas in the current study (including 9 known mother-calf pairs), demonstrated that all eight loci were inherited in a Mendelian fashion and were not sex-linked [27, 34, 43]. Comparisons of observed to expected genotypic frequencies using the Micro-Checker program (version 2.2.3) [46] for the four best sampled areas found only one of 32 tests consistent with null alleles (i.e., homozygote excess), and no evidence of large allelic dropout (0/32 tests) or scoring errors due to stuttering (0/32 tests).

**Table 1:** Sample sizes of beluga whales from fifteen geographic strata in the north Pacific: A resident population (Cook Inlet) and a separate small resident group (Yakutat Bay) in the Gulf of Alaska, six summering areas in the Bering, Chukchi, and Beaufort Seas, three summering areas in the Okhotsk Sea, and four locations along the northbound spring migration routes. Some of these strata have previously been identified as demographically separate management stocks based on mtDNA. While almost all samples were screened for microsatellites sample numbers for these nuclear markers indicate only those samples that yielded genotypes at six or more loci.

Region	Geographic Strata	Label in Figs 1 & 2	Stock	MtDNA	microsatellites	MtDNA	Total
				n	n	(Meschersky et al. 2013)	
Gulf of Alaska	Cook Inlet	*1*	*Cook Inlet*	133	78		133
	Yakutat Bay	*2*	*Cook Inlet*	8	8		8
Bering-Chukchi-Beaufort	Bristol Bay	*3*	*Bristol Bay*	140	129		140
	Norton Sound	*4*	*E*. *Bering Sea*	191	73		191
	Kotzebue Sound	*5*		119	64		119
	Kasegaluk Lagoon	*6*	*E*. *Chukchi Sea*	579	533		579
	Mackenzie Delta—Amundsen Gulf	*7*	*E*. *Beaufort Sea*	101	96		101
	Anadyr	*8*		46	44	37	83
Okhotsk	western Kamchatka	*9*		4	4	14	18
	Sakhalinskiy Bay	*10*		12	1	106	118
	Shantar—Udskaya Bay	*11*		10	10	46	56
BCB Migration	eastern Chukotka	A, B		10	9		10
	Little Diomede Island	C		11	10		11
	Point Hope	D		55	35		55
	Barrow—Kaktovik	E, F		16	13		16
	*cow-calf pairs			9	9		9
	All Locations			1444	1116	203	1647

*These represent the individuals from each of nine known cow-calf pairs sampled together that were screened but excluded from subsequent analysis.

### Data analysis

The amount and nature of mtDNA variation within the sequenced region were assessed by determining the number of variable sites and the number of unique haplotypes using MEGA 6.0 [47] software and by estimating both haplotypic (H) [48] and nucleotide (π) [49] diversity using Arlequin 3.5 software [50]. Estimation of genetic diversity (H_e_, H_o_ and number of alleles) and probabilities of identity (I) for nuclear loci was performed using Cervus 3.0 [51]. Tests for Hardy-Weinberg (H-W) and linkage equilibria were performed in Genepop 4.1 [52] with *P*-values estimated from 500,000 iterations of the data using the Mark chain method of Guo and Thompson [53]. An analysis of both mtDNA and nDNA data revealed that the populations were not in mutation-drift equilibrium (see [34]).

We used both frequency-based (F_st_) and distance-based statistics (Φ_st_, R_st_) to calculate the degree of genetic differentiation among spatial and temporal strata. Fixation indices were estimated using an analysis of variance framework [54, 55], and homogeneity tests based on 50,000 permutations of the data were performed in Arlequin. The model used for estimating the evolutionary distances between pairs of mtDNA sequences was simple pairwise differences. In the case of nuclear loci, genetic distances were based on stepwise mutation models. Confidence intervals for *F*_st_ and related parameters were estimated via 20,000 bootstraps of the multilocus nDNA data sets for each pairwise comparison in Arlequin. Mantel tests, based on 10,000 permutations of distance data, were conducted in Arlequin to assess the potential role of geographic distance in shaping the extent of genetic differentiation among geographic strata. Geographic distances were swim distances between the centroids of summering and wintering ranges estimated from satellite telemetry data [15, 16, 25, 31].

The Bayesian model-based clustering method, Structure 2.3.4 [56, 57], was used to infer population structure and the likely number of populations (*K*), and to assign individuals to population of origin. We conducted 28 distinct analyses with unique parameter settings: analyses were run both with and without admixture, with and without sampling location included as prior information, and they contained sample sets of varying size that were randomly sampled from the complete sample set using R (n = 15, 30, 50, 100, and all) that included individuals successfully genotyped at a range of loci (n ≥ 6 loci, n ≥ 7 loci, n = all 8 loci, see S1 Supporting information). For each parameter set we used a burn-in period of 50,000 iterations followed by 2x10^5^ iterations to collect data. Multiple runs (n = 5) were conducted for each value of *K* (n = 8) to ensure convergence for a total of 1,120 separate runs. The models of Hubisz et al. [58] use sampling location as prior information to reveal further underlying structure without risking detecting structure that is not present. To identify which individuals were likely immigrants (or descendants of recent immigrants) to their populations, we defined prior probabilities that each individual was an immigrant (*v* = 0.01–0.10) and incorporated information on the geographic origin of each sample before running the clustering analysis. We used Clumpak [59] to integrate results from multiple runs for different values of *K* and to implement methods for choosing *K*.

To further explore the origins of individual whales, we assigned individuals to populations based on estimated likelihoods of their genotype and/or mtDNA haplotype arising in each of the sampled populations under assumptions of random assortment of alleles and independence of loci using the programs Doh and whichrun 4.1 [60–62]. A log_10_ ratio (LOD) score of the most likely allocation to the second most likely of ≥1 (i.e., ratio of ≥10) denoted confidence in the assignment to a single population [61].

Differences in allelic diversity among populations can result in genotypes with higher calculated likelihoods in populations other than their source (i.e., nominal) population, even though the genotype may still be relatively common in the source population. In such cases an exclusion test is also required to ascertain likely population of origin. We thus examined the estimated likelihoods of genotypes *relative* to those of other genotypes *within* each baseline population. Using the Assignment Calculator in the Doh program [62] and assuming Hardy-Weinberg Expectations (HWE), 1,000 new individuals were generated from the gene pools of each population, and the natural log of the estimated probability of an individual’s genotype was compared to the likelihood distribution of the generated genotypes.

Dispersal patterns over ecological time scales were also investigated using the Bayesian approach of Wilson and Rannala [63] in the program BayesAss 1.3 (see S1 Supporting information for details). To investigate sex-bias in *genetic* dispersal (i.e., male-mediated gene flow on common wintering grounds or migration routes) we used the long-standing demographic and reproductive isolation between Cook Inlet and Arctic populations ([34], this study) to distinguish between the influences of *N*e, locus choice and gene flow on both marker types by comparing ratios of mean mtDNA to nDNA differentiation. Sex-bias in *actual* dispersal (i.e., individual transfer) was investigated by comparing levels of differentiation (F_st_) at mtDNA and levels of differentiation and average relatedness at nuclear markers (F_st_, F_is_, r, mAIc, vAIc) for males and females. The tests rely on sampling individuals post-dispersal in order to exclude the homogenizing effects of past immigration and interbreeding by either sex through the sampling of their offspring [64]. We assumed dispersal occurs primarily at the juvenile and sub-adult stage and so only adult cohorts were used. The randomization approach of Goudet et al. [64] was used to test whether there was significant contemporary sex-biased dispersal. Using Fstat 2.9.3 we compared observed differences to a null distribution based on 10,000 randomizations of the data.

We applied the Bayesian stock-mixture method of Pella and Masuda [65] to assess the population composition of migrating herds. This method incorporates uncertainty in the estimation of stock proportions of the ‘mixture’, or in this case migrant samples, and unlike conditional maximum likelihood methods improves stock determination by using information in the mixture sample to improve estimates of baseline relative frequencies. Using the program Bayes [66], we ran multiple chains with different starting parameters (including different prior stock proportions) and used the univariate statistic, the shrink factor, to monitor convergence of chains to the posterior probability. Analyses were conducted separately on mtDNA and nDNA data, and on mtDNA-and nDNA data combined.

Finally, because philopatry should promote high levels of relatedness among individuals sampled across years and generations within an area we tested this by comparing estimates of average relatedness and proportions of first and second-order relationships (based on nDNA) within and between years using the programs coancestry [67] and ml-relate [68] (See S1 Supporting information).

## Results

### MtDNA and nDNA diversity

A total of 1,444 individual beluga whales from throughout the North Pacific were screened for sequence variation within 410bp of the mtDNA control region and adjacent proline tRNA gene (Table 1, Figs 1 and 2). Twenty four variable sites were found, all of which were substitutions (23 transitions and 1 transversion). A total of 36 unique haplotypes were identified, 12 of which were represented by a single individual. To reduce sampling bias from non-random sampling of close kin, only one member from cow-calf pairs where both members were sampled together was included in subsequent analyses. Overall haplotypic diversity for the remaining 1,435 whales was high (H = 0.84) due to the large number of rare haplotypes while overall nucleotide diversity was moderate (π = 0.49%), indicating that the majority of haplotypes were phyletically closely related. A median joining network of the unique mtDNA haplotypes reflected this and was characterized by a series of star-like phylogenies with several rarer haplotypes radiating from a more common central haplotype, a pattern widely interpreted as indicative of ancient population expansions (see S1 Supporting information, S1 Fig).

A total of 1,116 individual whales were successfully screened for polymorphism at six or more microsatellite loci (Table 1). Fisher exact tests for HWE (i.e. 8 loci x 9 geographic strata) found 11 of 72 to be significant at α = 0.05. These deviations were partly due to combining three areas with low sample size into one Okhotsk area and were not consistent across areas or loci; involving 7 different loci and 8 separate locations, and no evidence of linkage was found among the 8 loci when tested across the major concentration areas (see S1 Supporting information). Average expected heterozygosity (*H*_e_) for the 8 loci ranged from 0.511 to 0.799 across the geographic strata (S1 Table). The number of alleles found per locus within an area ranged from 2 for locus CS415 in Yakutat Bay, Alaska to 16 for locus DlrFCB17 in the Mackenzie Delta-Amundsen Gulf, Canada, and the probabilities of identity (P(ID)) for each area were on the order of 10^−5^ to 10^−10^.

### Genetic differentiation and model-based cluster analysis

Substantial levels of mtDNA differentiation were observed among the three primary regions that beluga whales inhabit in the North Pacific for both the distance-based (Φ_st_ = 0.105–0.321) and frequency-based (*F*_st_ = 0.079–0.285) statistics (Table 2). All six major summer coastal concentration areas within the BCB region: Bristol Bay, Norton Sound, Kotzebue Sound, Kasegaluk Lagoon, Mackenzie-Amundsen, and Anadyr Bay were also substantially differentiated from each other (*F*_st_ = 0.106–0.507) (Table 3A), the only exception being the low level of mtDNA heterogeneity observed between Kotzebue Sound and Mackenzie-Amundsen (*F*_st_ = 0.022). The three primary summer concentration areas within the Sea of Okhotsk also displayed substantial mtDNA heterogeneity (*F*_st_ = 0.104–0.293). Comparing summer concentration areas and resident populations across the entire north Pacific, Cook Inlet and Bristol Bay were found to be the most distinct for mtDNA with average *F*_st_ values in excess of 0.39 and 0.44, respectively.

**Table 2:** Macrogeographic patterns of population differentiation in beluga whales in the north Pacific Ocean: (A) within mitochondrial DNA and (B) across eight microsatellite loci. Values for the frequency-based statistic, F_st_, are below the diagonal, values for the distance-based statistic, Φ_st_ (mtDNA) and R_st_ (nDNA), are above the diagonal. All P-values from homogeneity tests, based on 50,000 permutations, were significant at p<0.0001. n denotes sample size.

A. Mitochondrial DNA			
	Gulf of Alaska	BCB Seas	Sea of Okhotsk
*n =*	*141*	*1136*	*192*
Gulf of Alaska		0.163	0.321
Bering-Chukchi-Beaufort Seas	0.188		0.105
Sea of Okhotsk	0.285	0.079	
B. Microsatellites			
	Gulf of Alaska	BCB Seas	Sea of Okhotsk
*n =*	*86*	*939*	*15*
Gulf of Alaska		0.093	0.125
Bering-Chukchi-Beaufort Seas	0.049		0.112
Sea of Okhotsk	0.083	0.070	

**Table 3:** Population differentiation within mitochondrial DNA (A) and across eight microsatellite loci (B) in Pacific beluga whales. Values for the frequency-based statistic, F_st_, are below the diagonal. Values for the distance-based statistic, Φ_st_ (mtDNA) and R_st_ (nDNA), are above the diagonal. Corresponding *p*-values for homogeneity tests, based on 50,000 permutations, are represented by the following shading patterns: dark grey: p≤0.01, light grey: 0.01<p≤0.05, unshaded: p>0.05. Only strata with a sample size of n≥10 are reported, and the Beaufort Sea stratum is the Mackenzie and Point Hope strata combined (see text). Reported estimates of heterogeneity comprise the roughly two decade period from 1988–2010. See Table A and B in S7 Table for more details on temporal patterns of heterogeneity.

A. mitochondrial DNA															
	Summering Areas										Spring migration				
	GOA*	BCB						Okhotsk							
	Cook Inlet	Bristol Bay	Norton Sound	Kotzebue Sound	Kasegaluk Lagoon	Mackenzie-Amundsen	Anadyr Bay	Western Kamchatka	Sakhalin Bay	Udskaya Bay	Chukotka	Diomede	Point Hope	Barrow-Kaktovik	Beaufort Sea
*n =*	133	140	191	80	541	101	83	18	118	56	10	11	26	16	127
Cook Inlet		0.588	0.390	0.379	0.188	0.451	0.492	0.579	0.467	0.397	0.554	0.576	0.475	0.317	0.446
Bristol Bay	0.613		0.065	0.403	0.226	0.576	0.694	0.825	0.188	0.275	0.813	0.829	0.694	0.597	0.556
Norton Sound	0.361	0.106		0.243	0.176	0.423	0.514	0.617	0.182	0.173	0.572	0.608	0.466	0.294	0.420
Kotzebue Sound	0.346	0.400	0.179		0.210	0.056	0.151	0.481	0.319	0.236	0.162	0.222	0.068	-0.005	0.062
Kasegaluk Lagoon	0.242	0.436	0.297	0.222		0.289	0.347	0.417	0.184	0.207	0.321	0.367	0.294	0.161	0.296
Mackenzie-Amundsen	0.368	0.507	0.287	0.022	0.235	0.075		0.532	0.462	0.378	0.022	0.090	-0.016	0.028	-0.009
Anadyr Bay	0.453	0.659	0.422	0.123	0.323	0.064		0.501	0.543	0.417	0.144	0.180	0.074	0.120	0.073
western Kamchatka	0.439	0.723	0.419	0.304	0.308	0.328	0.346		0.566	0.315	0.644	0.633	0.535	0.500	0.524
Sakhalin Bay	0.332	0.279	0.120	0.143	0.235	0.218	0.336	0.293		0.192	0.540	0.578	0.469	0.350	0.464
Udskaya Bay	0.374	0.301	0.111	0.158	0.259	0.245	0.377	0.266	0.104		0.451	0.475	0.366	0.238	0.381
Chukotka	0.394	0.718	0.368	0.098	0.216	0.038	0.148	0.338	0.248	0.291		-0.051	-0.007	0.105	0.018
Diomede	0.367	0.690	0.334	0.103	0.219	0.077	0.186	0.289	0.210	0.250	-0.053		0.058	0.160	0.086
Point Hope	0.345	0.563	0.248	0.011	0.200	-0.004	0.102	0.278	0.162	0.189	0.020	0.045		0.030	-0.018
Barrow Kaktovik	0.356	0.584	0.236	-0.017	0.217	-0.009	0.079	0.339	0.176	0.203	0.087	0.104	-0.005		0.032
Beaufort Sea	0.353	0.476	0.273	0.020	0.228	-0.008	0.069	0.314	0.207	0.231	0.034	0.071	-0.011	-0.008	
B. microsatellites															
	Summering Areas										Spring Migration				
	GOA*	BCB						Okhotsk							
	Cook Inlet	Bristol Bay	Norton Sound	Kotzebue Sound	Kasegaluk Lagoon	Mackenzie-Amundsen	Anadyr Bay	Okhotsk Sea			Chukotka	Diomede	Point Hope	Barrow-Kaktovik	Beaufort Sea
*n =*	78	129	73	64	533	96	44	15			9	10	22	13	118
Cook Inlet		0.121	0.119	0.113	0.088	0.119	0.049	0.119			0.156	0.051	0.109	0.138	0.115
Bristol Bay	0.071		0.000	0.026	0.013	0.005	0.034	0.136			0.009	0.004	0.017	0.000	0.009
Norton Sound	0.051	0.014		0.033	0.013	0.010	0.024	0.155			0.028	-0.006	0.028	0.000	0.015
Kotzebue Sound	0.052	0.020	0.005		0.012	0.004	0.033	0.134			-0.007	-0.026	0.003	-0.003	0.005
Kasegaluk Lagoon	0.048	0.020	0.011	0.007		0.008	0.019	0.107			0.025	-0.026	-0.002	-0.005	0.007
Mackenzie—Amundsen	0.054	0.017	0.005	0.002	0.013		0.026	0.145			0.009	-0.022	-0.004	-0.011	-0.004
Anadyr Bay	0.036	0.013	-0.001	0.008	0.005	0.000		0.078			0.056	-0.023	0.031	0.033	0.027
Okhotsk Sea	0.083	0.086	0.080	0.078	0.074	0.071	0.030				0.230	0.094	0.116	0.187	0.136
Chukotka	0.063	0.003	0.006	0.003	0.009	-0.003	0.012	0.084				-0.006	0.026	-0.004	0.013
Diomede	0.041	0.016	-0.005	-0.007	-0.001	-0.001	-0.016	0.081			-0.004		-0.013	-0.019	-0.019
Point Hope	0.053	0.021	0.004	0.001	0.007	0.002	0.003	0.077			-0.001	0.001		-0.007	-0.007
Barrow—Kaktovik	0.037	0.012	0.005	-0.005	0.002	0.006	-0.010	0.063			-0.006	-0.002	-0.002		-0.009
Beaufort Sea	0.053	0.018	0.005	0.001	0.012	-0.004	0.000	0.071			-0.003	-0.001	-0.003	0.004	

*The three regions in the north Pacific are: GOA = Gulf of Alaska, BCB = Bering, Chukchi and Beaufort Seas, and Okhotsk Sea.

Overall, nDNA differentiation was lower than that of mtDNA but it too differed substantially among regions (Table 2B) and among most summer concentration areas and resident populations (Table 3B). As with mtDNA, Cook Inlet was the most distinct of all geographic strata for microsatellite variation (${\bar{F}}_{\mathrm{st}}$ = 0.056). No or very low levels of nDNA differentiation were observed between Norton Sound and Anadyr Bay, between Kotzebue Sound and Mackenzie-Amundsen and between Anadyr Bay and Mackenzie-Amundsen (Table 3B). Low sample size precluded analyses of nDNA heterogeneity within the Sea of Okhotsk. In a hierarchical AMOVA the proportion of total variation due to differentiation among regional groupings compared to among populations within groups was higher for nDNA than for mtDNA (see S1 Supporting information).

**Fig 3:**
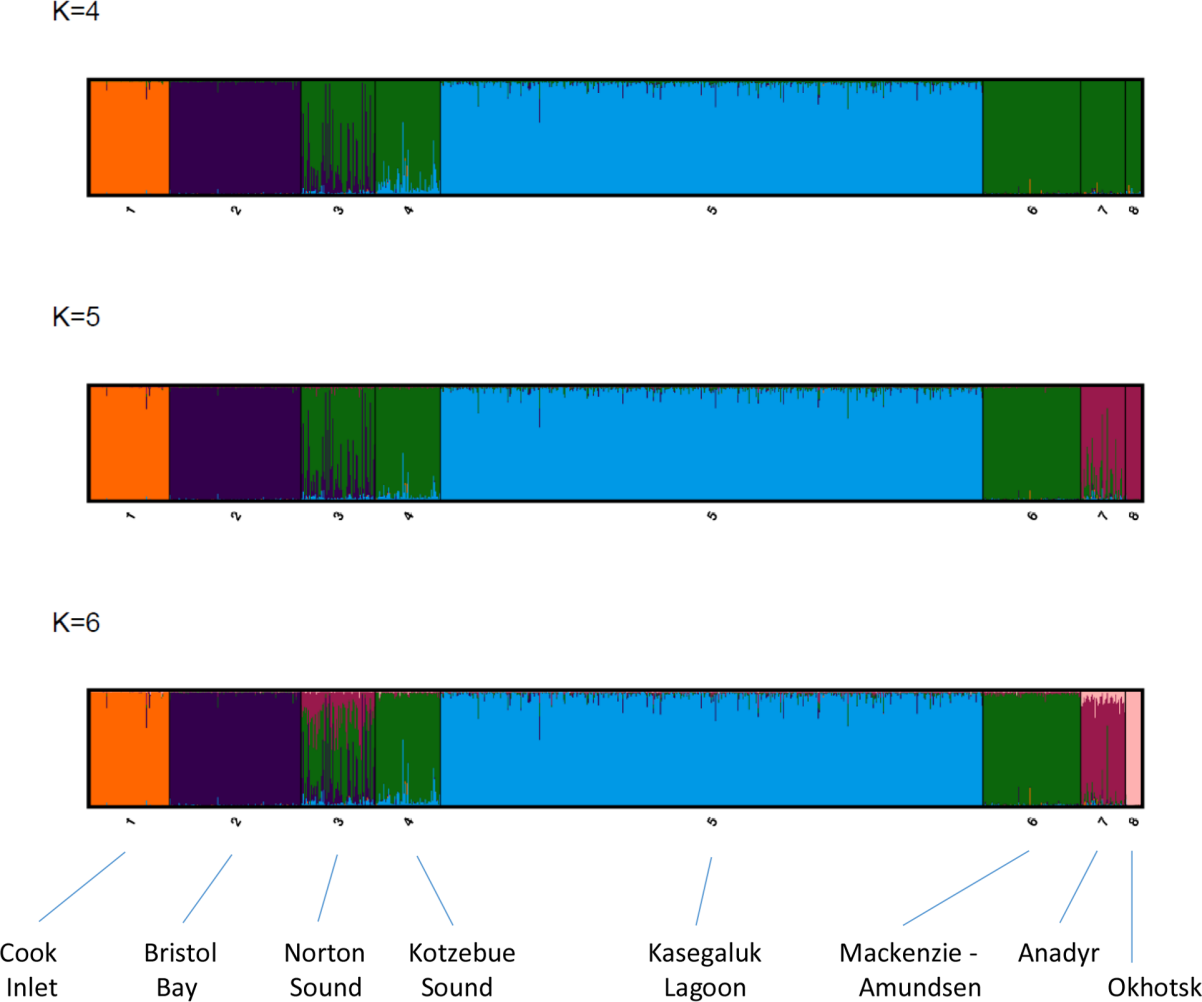
Summary plots generated in Clumpak of model-based cluster analysis of population structure in Pacific beluga whales using STRUCTURE 2.3.4. The major modes for *K* = 4 to 6 (based on five separate runs for each value of *K*) are presented for the analysis using prior sample group information and no admixture which revealed *K* = 5 clusters as the most likely (see panel 2). However, in a number of analyses *K* = 6 was the most or second-most likely resulting in the separation of Anadyr into a discrete cluster (see panel 3). Each genotyped individual is represented by a vertical line with estimated membership, Q, in each cluster denoted by different colors. The analysis was based on using all individuals (n = 1032) scored at 6 or more loci (n_loci_≥6).

There was widespread agreement across the various parameter settings used in the model-based cluster analysis (S2 Table). Using no prior information on the geographic origin of samples and no admixture among strata, the cluster analysis of genotypic data revealed that *K* = 2 populations was the most consistent with the data. The assignment of individuals revealed that these two genetically discrete population clusters corresponded to a combined Cook Inlet-Sea of Okhotsk stratum and the combined BCB stratum. Allowing for admixture, one population (*K* = 1) was the most likely, although the same geographic clustering of Cook-Okhotsk and BCB was still evident, especially for lower sample sizes (i.e., n = 30, n = 50), confirming that the primary genetic divergence in the data exists at a larger than regional scale. A re-analysis of the data incorporating prior information on sampling location detected additional structure. Most analyses, involving a range of sample sizes (n = 15, 30, 50, 100, all), loci (≥6, ≥7, 8) and levels of missing data (i.e., 0% - 4.9%), determined that five discrete population (*K* = 5) were most likely given the data (Pr (K|X ≈1.0, Fig 3, S2 Table). These population clusters corresponded to Cook Inlet, Bristol Bay, Kasegaluk Lagoon, Mackenzie-Amundsen and the Sea of Okhotsk. Norton Sound did not form a distinct population cluster, with all individuals assigned to the two populations corresponding to Mackenzie-Amundsen and Bristol Bay (Fig 3). Likewise, Anadyr Bay did not form a discrete population cluster in these analyses with individuals assigned to Mackenzie-Amundsen and the Sea of Okhotsk. However, Anadyr Bay did form a separate population in the few analyses where six populations (*K* = 6) were the most likely (n = 2/28) or in analyses where *K* = 6 was the second most likely (S2 Table). This is clearly evident in the Clumpak analyses where summary plots across multiple runs for each value of K reveal clear clustering of Anadyr in contrast to an absence of a distinct Norton Sound cluster (Figs 3 and S2). In all analyses Kotzebue Sound was part of the same cluster as Mackenzie-Amundsen.

**Fig 4:**
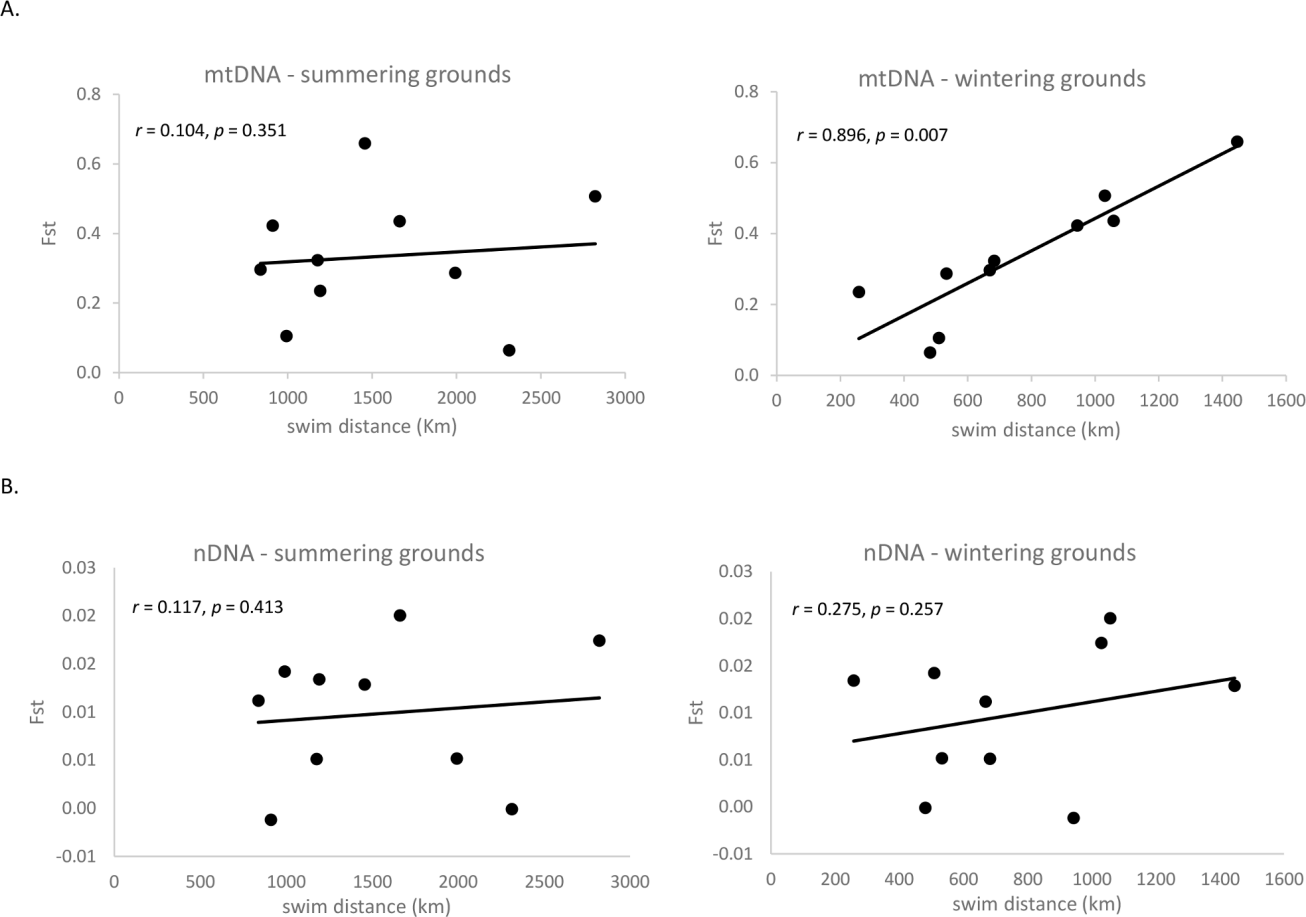
Mantel tests of the correlation between genetic differentiation (*F*_st_) and geographic distance among summering and wintering grounds of beluga whales in the Bering, Chukchi and Beaufort Seas, for both (A) mitochondrial DNA and (B) microsatellite markers. Test *p* values are based on 10,000 permutations of the distance data.

Mantel tests revealed positive correlations between genetic differentiation and geographic distance within the BCB region (Fig 4). Stronger correlations with genetic heterogeneity were found among wintering areas (r_mtDNA_-*F*_st_ = 0.9, *p* = 0.007) compared to summering areas (r_mtDNA_-*F*_st_ = 0.1, p = 0.351) and for mtDNA (r = 0.1–0.9) compared to nDNA (r = 0.12–0.27) (S3 Table).

### Seasonal migration

**Fig 5:**
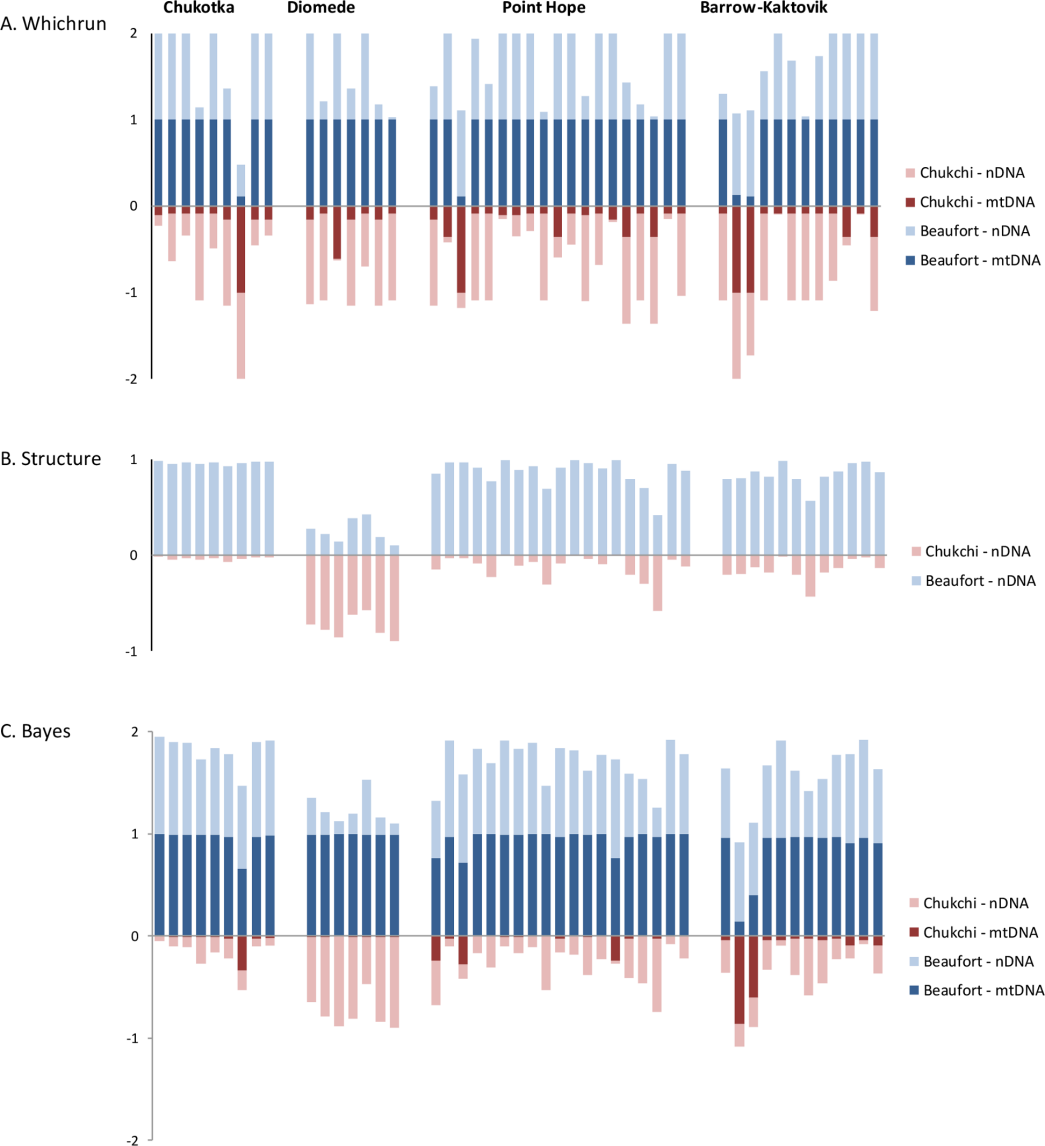
The likely population of origin of beluga whales on spring migration sampled at four locations in the Bering, Chukchi and Beaufort Seas. Individual assignments are represented as the relative height of stacked bars to either of two baseline populations, the eastern Beaufort Sea (blue) or eastern Chukchi Sea (red) for mtDNA (dark shading) and nDNA (light shading). A: Maximun Likelihood assignments in Whichrun. B: Baysian assignments using prior sampling information and admixture models in Structure 2.3.4. C: Mixed-stock assignments in Bayes. Only individuals with complete mtDNA-nDNA profiles are shown. See Table 4 for more details.

Maximum likelihood assignment tests were performed on four geographic groups of whales sampled on northbound spring migration (near Point Hope, eastern Chukotka, and Little Diomede Island) or during summer and early fall outside traditional concentration areas (near Barrow and Kaktovik) to determine population of origin (Table 1, Fig 2). Of the likely migration destinations, Whichrun assigned the majority of Point Hope whales (95.4%) and whales from the other three areas (85.7%) to the Mackenzie-Amundsen concentration area (i.e. eastern Beaufort Sea) for mtDNA (Table 4). Individual likelihoods of these whales based on nuclear genotypes were also higher for this concentration area over the Kasegaluk Lagoon concentration area (i.e., eastern Chukchi Sea) with many having combined mtDNA-nDNA LOD scores > 1 for Mackenzie-Amundsen (n = 23/45) (Table 4). Breaking assignments out by marker type revealed that mtDNA had a greater influence than any other locus on cumulative likelihoods (Fig 5A).

Assessing population origins of groups of migrating whales by using sample group information in Structure, the model-based clustering method (nDNA only) assigned all the Chukotka, Point Hope and Barrow-Kaktovik whales to the Mackenzie–Amundsen summering concentration in the eastern Beaufort (Table 4, Fig 5B). By contrast, the majority of the whales migrating past Diomede had higher inferred membership in, or ancestry (Q) from, Kasegaluk Lagoon in the eastern Chukchi Sea.

**Table 4:** The likely population of origin of beluga whales on spring migration sampled at four locations in the Bering, Chukchi and Beaufort Seas. Maximum likelihood assignments to two candidate populations, the eastern Chukchi Sea (Kasegaluk Lagoon) and the eastern Beaufort Sea (Mackenzie-Amundsen), were conducted in Whichrun and are reported both as likelihood ratios (P(n)/P(max)) and the Log of these ratios (LOD) for each individual. Bayesian assignments, using prior sample group information (i.e., LOCPRIOR models), were made using STRUCTURE and are reported as the estimated ancestry, Q, in Clusters 1 (Chukchi) and 2 (Beaufort). Assignments of individual migrants were also estimated using the stock-mixture method in BAYES, summarized here as the proportion of times, *P*, an individual was assigned to each baseline population.

sampled in	Lab ID	Whichrun					Structure		Bayes			
		P(n)/P(max)				LOD†	Q		*P*			
		nDNA		mtDNA			nDNA		nDNA		mtDNA	
		Chukchi	Beaufort	Chukchi	Beaufort		Chukchi	Beaufort	Chukchi	Beaufort	Chukchi	Beaufort
east Chukotka	23613	0.11	1	0.03	1	2.53	0.25	0.75	0.05	0.95	0	1
east Chukotka	23614	0.31	1	0.06	1	1.75	0.28	0.72	0.09	0.91	0.01	0.99
east Chukotka	23615	0.57	1	0.06	1	1.49	0.28	0.72	0.10	0.90	0.01	0.99
east Chukotka	23617	1	0.37	0.06	1	0.81	0.32	0.68	0.26	0.74	0.01	0.99
east Chukotka	23619	0.67	1	0.06	1	1.42	0.29	0.71	0.15	0.85	0.01	0.99
east Chukotka	23620	1	0.29	0.18	1	0.20	0.32	0.68	0.19	0.81	0.03	0.97
east Chukotka	23621	1	0.72	1	0.13	-1.02	0.29	0.71	0.19	0.91	0.34	0.66
east Chukotka	4674	0.30	1	0.18	1	1.75	0.17	0.84	0.07	0.93	0.03	0.97
east Chukotka	4675	0.19	1	0.18	1	1.65	0.17	0.83	0.07	0.93	0.03	0.97
Little Diomede	10262	1	0.36	0.18	1	0.29	0.56	0.44	0.64	0.36	0.01	0.99
Little Diomede	17121	1	0.18	0.06	1	0.49	0.58	0.42	0.78	0.22	0.01	0.99
Little Diomede	23301 *	1	0.49	-	-	-	0.60	0.40	0.84	0.16	-	-
Little Diomede	34140	1	0.13	0.09	1	0.15	0.60	0.40	0.88	0.12	0	1
Little Diomede	34141 *	0.11	1	-	-	-	0.44	0.56	0.26	0.74	-	-
Little Diomede	34142	1	0.30	0.18	1	0.22	0.54	0.46	0.80	0.20	0	1
Little Diomede	34574	0.53	1	0.06	1	1.52	0.52	0.48	0.46	0.54	0.01	0.99
Little Diomede	56711	1	0.36	0.18	1	0.30	0.59	0.41	0.83	0.17	0.01	0.99
Little Diomede	56712	1	0.06	0.06	1	0.01	0.62	0.38	0.89	0.11	0.01	0.99
Little Diomede	56713 ^¶^	-	-	0.18	1	-	-	-	-	-	0.01	0.99
Little Diomede	61098 ^¶^	1	0.33	-	-	-	0.55	0.45	0.71	0.29	-	-
Point Hope	2328	1	0.22	0.18	1	0.09	0.22	0.78	0.44	0.56	0.24	0.76
Point Hope	2329	0.10	1	0.39	1	1.42	0.17	0.83	0.07	0.94	0.03	0.97
Point Hope	2330	0.11	1	1	0.13	0.08	0.17	0.83	0.14	0.86	0.28	0.72
Point Hope	3791	1	0.78	0.06	1	1.14	0.19	0.81	0.17	0.83	0.00	1
Point Hope	4096 *	1	0.50	-	-	-	0.23	0.77	0.30	0.70	-	-
Point Hope	4097	1	0.69	0.06	1	1.08	0.24	0.76	0.31	0.69	0	1
Point Hope	4098	0.02	1	0.03	1	3.22	0.15	0.85	0.09	0.92	0.01	0.99
Point Hope	4432	0.21	1	0.03	1	2.27	0.21	0.80	0.16	0.84	0.01	0.99
Point Hope	7650	0.16	1	0.06	1	2.05	0.19	0.81	0.11	0.89	0	1
Point Hope	7654	1	0.07	0.06	1	0.10	0.25	0.75	0.53	0.47	0	1
Point Hope	7655 *	0.63	1	-	-	-	0.20	0.80	0.13	0.87	-	-
Point Hope	7656	0.37	1	0.39	1	0.84	0.20	0.80	0.13	0.87	0.03	0.97
Point Hope	7657	0.03	1	0.06	1	2.80	0.16	0.84	0.18	0.82	0	1
Point Hope	7658	1	0.30	0.03	1	1.07	0.26	0.74	0.37	0.63	0.01	0.99
Point Hope	7659	0.26	1	0.06	1	1.82	0.20	0.80	0.23	0.77	0	1
Point Hope	7660	0.09	1	0.18	1	1.78	0.15	0.85	0.03	0.97	0.24	0.76
Point Hope	7661 *	1	0.12	-	-	-	0.23	0.77	0.56	0.45	-	-
Point Hope	7662	1	0.80	0.39	1	0.31	0.23	0.77	0.38	0.62	0.03	0.97
Point Hope	7663	1	0.29	0.06	1	0.70	0.25	0.75	0.46	0.54	0	1
Point Hope	7665	1	0.02	0.39	1	-1.30	0.33	0.67	0.71	0.29	0.03	0.97
Point Hope	7666	0.07	1	0.06	1	2.38	0.18	0.82	0.08	0.92	0	1
Point Hope	7667	0.43	1	0.06	1	1.61	0.21	0.79	0.22	0.78	0	1
Point Hope	7913 ^¶^	-	-	0.18	1	-	-	-	-	-	0	1
Point Hope	7914 *^¶^	-	-	-	-	-	-	-	-	-	-	-
Point Hope	17863 ^¶^	-	-	0.06	1	-	-	-	-	-	0	1
Point Hope	26239 ^¶^	-	-	0.03	1	-	-	-	-	-	0	1
Barrow-Kaktovik	7648	1	0.28	0.06	1	0.69	0.23	0.77	0.32	0.68	0.04	0.96
Barrow-Kaktovik	7649	1	0.44	1	0.11	-1.31	0.23	0.77	0.22	0.78	0.86	0.14
Barrow-Kaktovik	7651	1	0.46	1	0.13	-1.21	0.21	0.79	0.29	0.71	0.60	0.40
Barrow-Kaktovik	7652	0.69	1	0.06	1	1.41	0.22	0.78	0.29	0.71	0.04	0.96
Barrow-Kaktovik	7653	0.07	1	0.06	1	2.41	0.16	0.84	0.05	0.95	0.04	0.96
Barrow-Kaktovik	7923	1	0.70	0.06	1	1.09	0.23	0.77	0.35	0.65	0.03	0.97
Barrow-Kaktovik	7924	1	0.04	0.06	1	-0.19	0.30	0.70	0.55	0.45	0.03	0.97
Barrow-Kaktovik	7925	1	0.11	0.06	1	0.27	0.23	0.77	0.42	0.58	0.04	0.96
Barrow-Kaktovik	8399 *	0.08	1	-	-	-	0.16	0.84	0.05	0.95	-	-
Barrow-Kaktovik	14314	0.81	1	0.06	1	-	0.21	0.79	0.20	0.80	0.03	0.97
Barrow-Kaktovik	25490 ¶	-	-	1	0.09	-	-	-	-	-	0.86	0.14
Barrow-Kaktovik	27494	0.40	1	0.39	1	1.47	0.18	0.82	0.19	0.81	0.09	0.91
Barrow-Kaktovik	7922	0.02	1	0.06	1	3.01	0.16	0.84	0.04	0.96	0.04	0.96
Barrow-Kaktovik	37535	1	0.33	0.39	1	-0.07	0.22	0.78	0.28	0.72	0.09	0.91

^†^LOD: Log10 of the ratio of the second most likely population for mtDNA and microsatellites combined. Positive values indicate assignments to the Beaufort (Mackenzie), negative values to the eastern Chukchi

^*^Individual whose haplotype was not in the baseline samples.

¶ Individual with incompletely scored genotype or haplotype.

**Fig 6:**
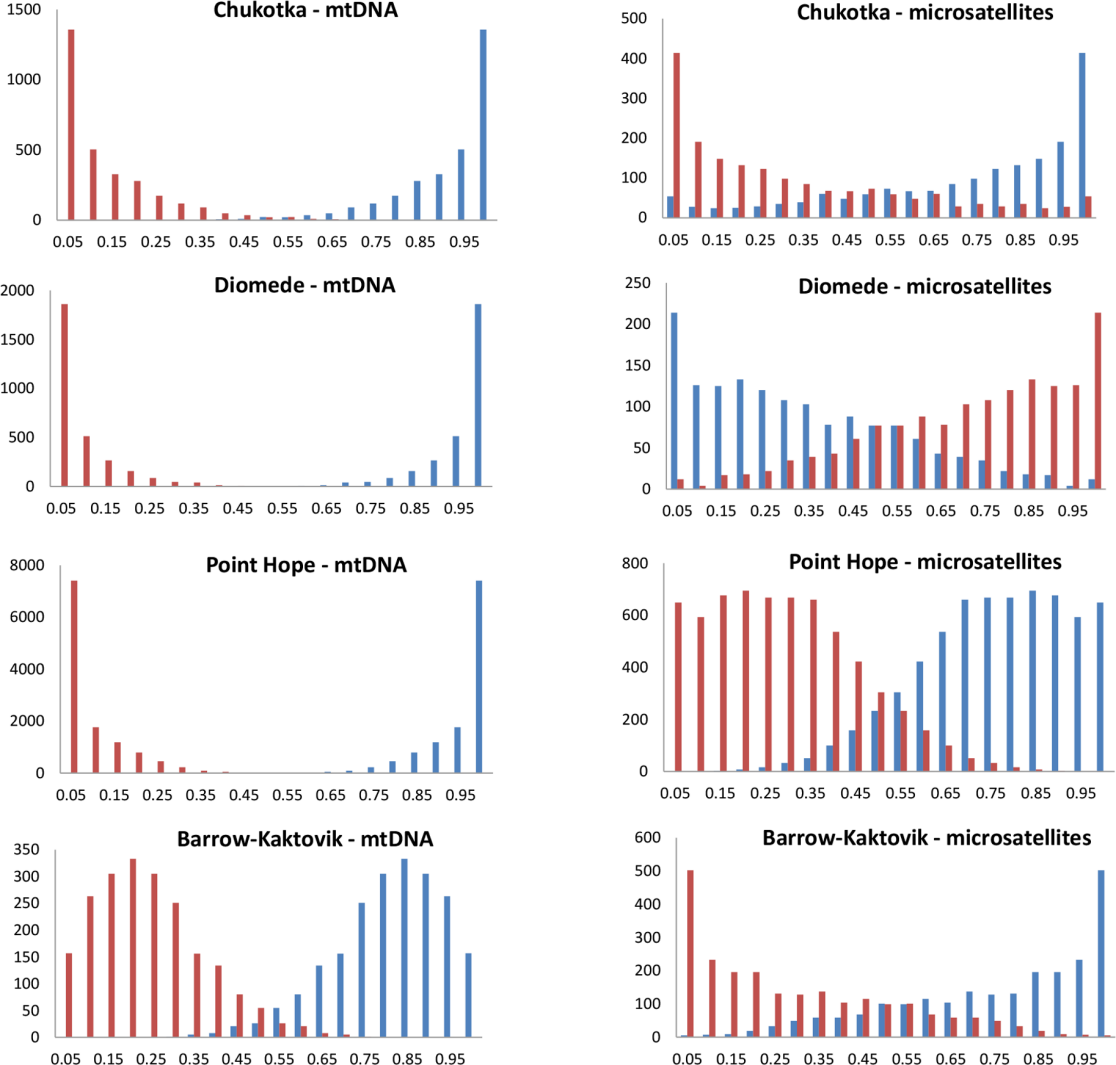
The probability distribution of population proportions of groups of beluga whales sampled on northbound migration in spring. Stock-mixture analysis was conducted in Bayes with the eastern Beaufort Sea (blue) and the eastern Chukchi Sea (red) as baseline populations and the migrating groups as the potential ‘mixtures’. The ordinate axis indicates the number of runs.

The stock-mixture method, Bayes, estimated population proportions and thus likelihoods of migrating groups being of mixed origin, and assigned individual migrants probabilistically to baseline populations. For all runs, estimated shrink factors were close to one (1.00–1.01) indicating convergence of multiple chains generated from different starting proportions to the posterior density. Viewing the mtDNA results, groups of migrating whales from all four strata had very high likelihoods (median = 0.80–0.97) of originating from the eastern Beaufort and correspondingly low likelihoods of coming from the eastern Chukchi Sea (Fig 6, S4 Table). Only belugas sampled near Barrow had moderate likelihoods of possible mixed composition. The microsatellite results also found high population proportions from the eastern Beaufort for Chukotka, Point Hope and Barrow-Kaktovik, although the density distributions exhibited greater overlap compared to mtDNA. In contrast to the mtDNA findings, the Diomede whales had the highest likelihood of coming from the eastern Chukchi Sea. Individual assignments for each marker type affirmed the group findings that herds migrating past Chukotka and Point Hope in spring were unlikely to be a mix of Beaufort and Chukchi Sea whales, with almost all assigned to the Beaufort at P>0.9 for mtDNA and P>0.6 for microsatellites (Fig 6, S4 Table). Assignments differed dramatically among marker types for Diomede with Bayes finding high microsatellite likelihoods that most of the Diomede whales (8/10) were from the Chukchi but high mtDNA likelihoods that the Diomede whales hailed from the Beaufort (Fig 5C). Finally, while both nDNA and mtDNA assigned most of the Barrow-Kaktovik whales to the Beaufort, some individuals were assigned to the Chukchi Sea based on mtDNA data (Fig 5C).

While the assignment of separate groupings into the same ‘population cluster’ is not in itself proof of random mixing, these findings and the absence of mtDNA and nDNA differentiation, in combination with the timing of the northbound migration past Point Hope and the arrival of beluga whales at the Mackenzie Delta and Amundsen Gulf, indicate that the whales from both locations are part of the same population, the eastern Beaufort Sea population. It is for this reason that analyses of dispersal and temporal patterns (see below) were conducted with the Point Hope and Mackenzie-Amundsen whales combined in a single eastern Beaufort Sea stratum.

### Dispersal

Genetic estimates of recent dispersal rates using BayesAss indicated negligible immigration (m≤0.004) into Cook Inlet from other populations (S5 Table). By contrast, uncertainty over estimates among summering concentrations within BCB suggests that, unlike the comparisons between Cook Inlet and BCB, there was not enough information in the data to estimate rates of dispersal within BCB over such a short time frame (a few generations) using this method (see S1 Supporting information).

In the assessment of sex-biased gene flow through comparison of relative ratios of nDNA to mtDNA differentiation, estimates of microsatellite heterogeneity (*F*_st_) for those pair-wise comparisons involving Cook Inlet were, on average, 7.1 times lower than corresponding estimates for mtDNA (Table 3). By contrast, mean microsatellite differentiation among the four BCB summering areas was 22.9 times lower than corresponding mtDNA estimators. Similar results were found with distance-based estimators. Two exceptions to this general pattern are noteworthy. Firstly, the ratio for Bristol Bay-Norton Sound is low (*F*_st_ ratio = 7.3) due to the high frequency of mtDNA Hap#5 in both areas. Secondly, the ratio for Norton Sound-Mackenzie is very high (*F*_st_ ratio = 55.6) due to the very low levels of microsatellite differentiation observed between these two areas (Table 3), a finding also reflected in the clustering analysis (Fig 3).

Assessment of contemporary sex-biased dispersal through comparisons of genetic heterogeneity and relatedness for post-dispersal age cohorts was possible for the eastern Beaufort and Chukchi Seas for the decade 1988–1997. MtDNA differentiation among adult (≥350 cm standard length) males, though substantial, was lower than differentiation among adult (≥300cm standard length) females (*F*_*s*t males_ = 0.218 v. *F*_st females_ = 0.235, Table 5A). Similarly, using fstat allelic frequency differences at nuclear markers between the eastern Beaufort and eastern Chukchi were significantly lower for adult males compared to adult females (*F*_st males_ = 0.010 v. *F*_st females_ = 0.022, *p* = 0.043, Table 5A), while estimated average relatedness within each area was significantly lower for males compared to females (*r*_males_ = 0.0204 v. *r*_females_ = 0.0419, *p* = 0.042). These sex differences were more pronounced in large (i.e., older) adults (Table 5B).

**Table 5:** Genetic differentiation (*F*_st_) between post-dispersal age cohorts of beluga whales from the eastern Chukchi (Kasegaluk Lagoon) and the Beaufort Seas (Mackenzie-Amundsen). Pairwise estimates for mtDNA are below the diagonal and for microsatellites above the diagonal. Analyses were conducted on all adults (A) and on all large, and presumably older, adults (B). Sample sizes (n) for the mtDNA comparisons are in column 2 and for microsatellites in row 3. Estimates of age were based on the number of growth layer groups (GLGs) in sectioned teeth.

A. *F*_st_ - adults					
		Chukchi males	Chukchi females	Beaufort males	Beaufort females
	*n =*	59	59	62	37
Chukchi males	65		-0.002	0.010	0.012
Chukchi females	62	-0.004		0.014	0.022
Beaufort males	67	0.218	0.268		-0.001
Beaufort females	40	0.182	0.235	0.003	
B. *F*_st_—large adults
			Chukchi males	Chukchi females	Beaufort males	Beaufort females
			*n =*	25	33	45	22
	Chukchi males	29		0.001	0.007	0.006
	Chukchi females	35	0.054		0.014	0.031
	Beaufort males	47	0.118	0.278		0.001
Beaufort females	23	0.117	0.287	-0.023	

Adult males: ≥ 350 cm standard length.

Adult females: ≥ 300 cm standard length.

Large/older adult males: ≥ 400 cm (Chukchi) ≥ 415 cm (Beaufort) and/or ≥ 25 GLGs.

Large/older adult females: ≥ 350 cm and/or ≥ 25 GLGs.

Patterns of recent dispersal were also characterized using individual-assignment methods. Using four likelihood and Bayesian inference methods very few individuals were identified as possible migrants or of migrant ancestry (S6 Table, see S1 Supporting information). Of all the individual whales identified as possible migrants or of having recent migrant ancestry, the majority (74%) were adult males (S6 Table). While the number of males sampled in each population tends to exceed that of females [22], comparison of the sex ratio for the most thoroughly sampled population, the eastern Chukchi Sea (M:F– 1.56:1) [22], to that for likely migrants or migrant descendants identified here (M:F– 5.67:1) revealed a significant male bias (χ^2^ = 4.77, *p* = 0.03).

### Temporal and kinship analysis

Temporal comparisons on decadal scales revealed almost no change in the geographic patterns of mtDNA and nDNA variation for almost all geographic strata over ecological time frames. Mean mtDNA differentiation among time periods (i.e., 1980s, 1990s, 2000s, etc.) within each area did not differ significantly from zero (${\bar{F}}_{\mathrm{st}}$ = 0.017 ± 0.1, *p* >0.05; Table A in S7 Table), and was significantly lower than corresponding mean values among areas (${\bar{F}}_{\mathrm{st}}$ = 0.308 ± 0.15; t-test: *p* = 0.0003). Results were similar for nDNA (Table B in S7 Table), while Structure assigned whales from each summering concentration area sampled in different time periods into the same population clusters (S4 Fig). As with the original combined dataset, Norton Sound exhibited low differentiation (*F*_*st*_) from, and clustered with, both Bristol Bay and the eastern Beaufort Sea. The one exception was Kotzebue Sound, where substantial mtDNA differentiation (*F*_st_ = 0.12–0.31) was observed between beluga whales sampled in the 1980s prior to the population decline and whales sampled in the 1990s and 2000s (Table A in S7 Table). No nDNA data were available for the 1980s as the DNA was recovered by ancient DNA methods from teeth and dried tissue.

**Fig 7:**
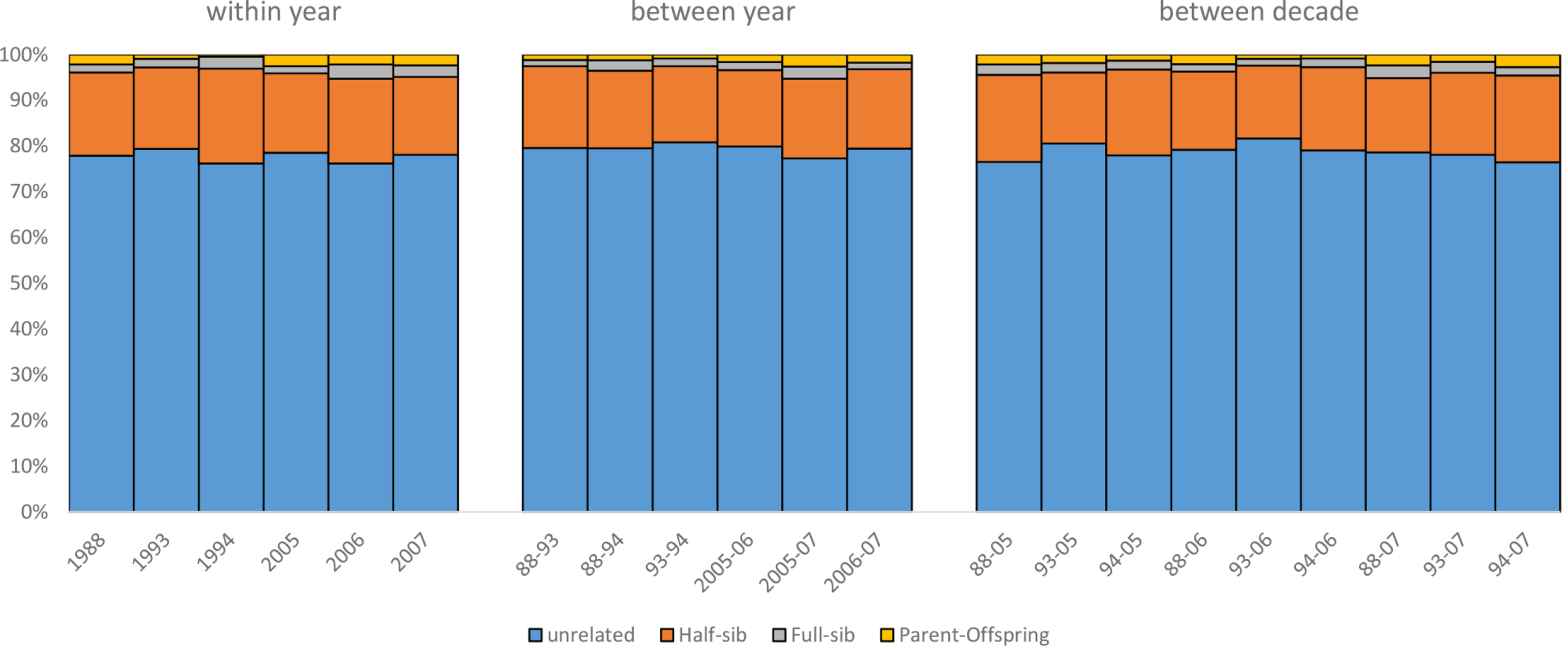
The proportion of pairwise genealogical relationships estimated for beluga whales sampled within and between years across two decades near Kasegaluk Lagoon, Alaska. Maximum likelihood estimates of four relationship categories were estimated from genotypic data using the program ml-relate. The stacked bars represent the proportions of distantly/unrelated individuals to closely related individuals (i.e., parent-offspring, full-sib and half-sib or equivalent) for a subset of the 20-year data set comprising the first three years (1988, 1993, 1994) and the last three years (2005, 2006, 2007).

**Fig 8:**
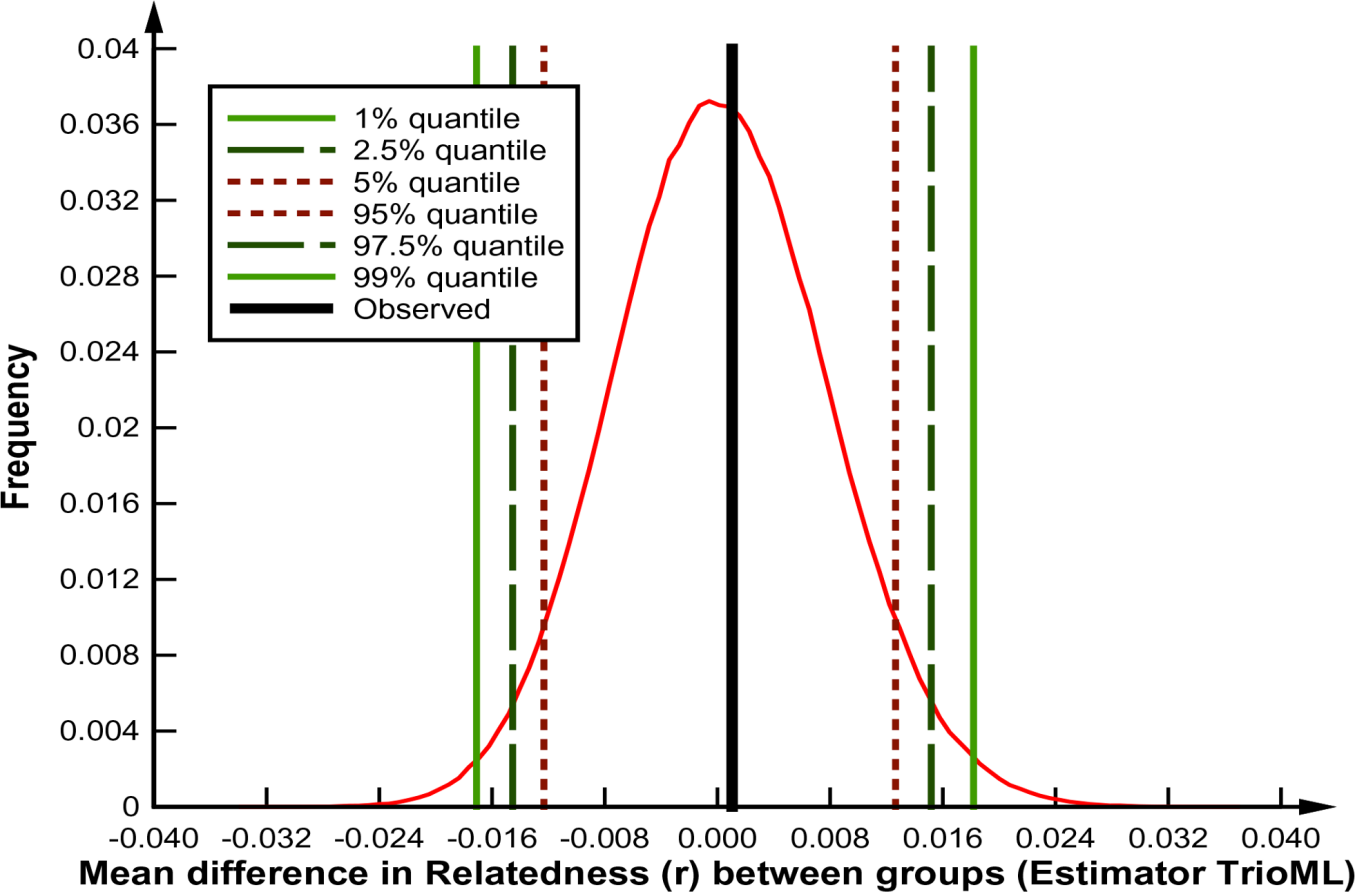
Test of differences in mean relatedness ($\bar{r}$) among beluga whales within a single year compared to $\bar{r}$ between whales from two different years for the same summering ground using coancestry. The graph depicts results for Kasegaluk Lagoon 1988 compared to 1988–2007 using the ML estimator TrioML of Wang (2007). If the observed difference (black line) falls outside the 90% (dotted lines), 95% (dashed lines), and 99% (green solid lines) confidence intervals from the bootstrap analysis distribution the difference is adjudged to be significant.

The relatedness analyses found substantial numbers of closely related individuals within groups of whales sampled together at the Kasegaluk Lagoon summer aggregation area (Fig 7). The analysis also found that substantial numbers of closely related belugas, including likely parent and offspring pairings, were sampled across years within summering concentration areas. The coancestry analysis revealed that the mean estimate of pairwise relatedness, $\bar{r}$, within years did not differ significantly from $\bar{r}$ across years within the same summering concentration for both moment and maximum likelihood (ML) estimators. This was the case for sample years up to 20 years apart (Fig 8). Inferring pairwise relationships from ML estimates of *r* in ml-relate revealed that the proportions of close relatives (i.e., parent-offspring, full-sib, half-sib or equivalent) to distant or unrelated individuals within a given year was consistently on the order of 20%:80% (*G* = 0.2–1.8, *p*>0.5), and this within-year ratio was not found to differ to relationship ratios across years, even for comparisons separated by up to 20 years (*G* = 0.2–2.9, *p*>0.5, Fig 7).

## Discussion

The study provided compelling evidence for widespread natal philopatric behavior of beluga whales not just to summering concentration areas but to entire migratory circuits within regions where there are few geographic barriers to dispersal enabling distinct subpopulations to overlap in space and time. Closely related whales were found to aggregate together at coastal summering areas each year, and close kin were documented at the same summering sites up to twenty years apart. We found clearer evidence of sex-biased dispersal than previous studies, and documented stability in migration and dispersal behavior over ecological (i.e., decadal) time frames with notable exceptions. A much expanded nDNA dataset revealed a pattern of limited interbreeding among many distinct subpopulations within the BCB region where the spatial proximity of winter ranges and spring migration routes appears to have a greater influence on the degree of both individual and genetic exchange than summer ranges. This study supports recent satellite telemetry evidence of a series of mostly distinct, if overlapping, wintering areas within the BCB region [31], and contrasts with earlier genetic studies [27] that suggested a single common wintering area and panmixia across all subpopulations within the Bering, Chukchi and Beaufort Seas.

Extensive spatial and temporal sampling and thorough individual-based data analysis provided a more detailed insight into the migration patterns, areas of mixing, dispersal behavior and gene flow of this long-lived, highly vagile species than previously possible. The model-based cluster analysis and assignment tests were greatly improved by larger sample sizes and locus number, informed run assumptions, and the inclusion of all potential population clusters. The limited information content in the microsatellite data within regions severely limited the utility of the program BayesAss to estimate recent rates of dispersal, something recognized by a number of other recent studies [34, 69]. Below we discuss the investigation’s findings and their implications for beluga whale behavioral ecology and management in more detail.

### Demographic history

Phylogeographic patterns of differentiation at nDNA and mtDNA markers among beluga whale populations from the Gulf of Alaska, BCB, and Sea of Okhotsk regions in the Pacific likely reflect long standing patterns of restricted gene flow and dispersal. This was especially so for the Cook Inlet population in the Gulf of Alaska confirming that this small, geographically isolated population is both reproductively and demographically isolated from all other beluga whale populations, and likely has been over evolutionary time frames (see S1 Supporting information). These basin-wide findings expand on earlier regional-level studies [32–35, 39] and are supported by recent satellite telemetry studies that have mapped winter as well as summer movements [17, 31]. Together, these investigations indicate that the Alaska and Kamchatka Peninsulas have been effective barriers to dispersal and gene flow over time. Projections for continued climate warming across the Arctic [70] in concert with inherent natal homing tendencies of beluga whales (see below) will likely act to maintain distinct regional populations in the future.

The sequence by which discrete geographic strata emerged as distinct population clusters in the cluster analysis may also reveal something about their origins. Bristol Bay and Kasegaluk Lagoon emerged as discrete population clusters early on in the various cluster analyses, that is, at low values of K (see Fig 3, S2 Table) while most other strata within the BCB region as well as the small Okhotsk cluster did not differentiate out until later (i.e., higher K). Multiple factors may contribute to such findings (see S1 Supporting information) but this may reflect an early divergence of these populations within the BCB region and possible separate glacial refugia for these two populations.

### Philopatry, group structure and migratory culture

The substantial mtDNA differentiation we detected for both sexes among summering concentration areas within the Bering, Chukchi, Beaufort, and Okhotsk Seas likely indicates limited dispersal among these areas and a probable long established pattern of female-mediated philopatry to summer migration destinations. Although this has become an almost universal interpretation for such patterns of mtDNA variation for migratory species [7, 32, 35, 39, 71–77], and see [4], it has rarely been explicitly tested. It relies on the assumptions of a simple drift-dispersal model to explain predominantly frequency-based (*F*_st_) differentiation. However, multiple factors can complicate interpretations of mtDNA subdivision in terms of contemporary dispersal patterns: local populations may not be in equilibrium, sampling may be inadequate, and movement patterns in migratory species may change across years and generations (see below). Using large sample sizes collected across many years we identified very low numbers of females and males as likely migrants or of migrant ancestry and recorded consistent patterns of mtDNA heterogeneity among most areas over decades of sampling. With age of first reproduction estimated at 8.3yr for Nearctic belugas [22] this time series likely spanned multiple generations and supports philopatry to natal population or summering group as a dominant trait in beluga whales. The discovery that substantial numbers of closely related whales, including parent-offspring pairings, were sampled across years, and even decades, within summering areas is direct proof of natal homing.

A recent study summarizing findings from multiple satellite telemetry investigations revealed that beluga whales from separate summering grounds across the BCB region return to somewhat geographically discrete wintering areas in the northern Bering Sea [31] (Fig 2). Our genetic findings viewed alongside these winter movement and habitat use data indicate that philopatry in beluga whales may extend to entire migratory circuits including migration routes, staging areas, molting sites, summering locations and wintering areas, which in turn promotes the emergence and maintenance of demographically distinct subpopulations regardless of the extent of seasonal overlap (Fig 2) or level of interbreeding (see below).

Natal philopatry has been documented or inferred in a wide range of migratory species and long-distance travelers, including birds [2, 78], fish [79], reptiles [76], and mammals [80]. In species where there is an extended period of postnatal care or association with parents it has been postulated that this homing behavior can entail migratory culture where migratory routes and destinations are learned from parents [1, 6, 7]. Whitehead [4] recently declared that the evidence for culture in non-humans is rare and that documenting cultural stability (in non-humans) is difficult. Furthermore, while genetic differentiation may reflect the faithful transmission of migratory culture [4], it is not direct evidence of it.

Colbeck et al. [81] recently found that groups of closely related beluga whales often migrate together in the Hudson Bay region of Canada and posited that this could facilitate the learning of migration routes. Matthews and Ferguson [82] confirmed that beluga whale calves are dependent on their mothers for two or more years, and thus multiple migratory cycles. Such findings when viewed in conjunction with our findings of limited dispersal among areas, and substantial numbers of close kin not only within herds and years at discrete summering areas but also across decades at those locations indicate the potential for lifelong associations of closely related individuals and is compelling evidence for culturally inherited fidelity to migration destinations in beluga whales that may extend to entire migratory circuits.

The annual return of beluga whales to traditional locations at specific times of year is likely an adaptation to the non-uniform distribution of resources across the north Pacific and the seasonal predictability of those resources, including food, molting sites, and calving and breeding areas. The configuration of the migratory circuit of each beluga whale population or stock (Fig 2), and thus their degree of overlap, has likely also been shaped by physical factors that directly shape migration pathways (e.g., sea ice, geography) and by historical factors such as the routes of postglacial colonization. Any shift in environmental parameters could presumably disrupt migration routes and seasonal habitat use patterns (see below), and/or the level of philopatry to those demographically distinct circuits. A recent study revealed that beluga whale movements are resilient to inter-annual variation in sea ice conditions [83] which may be related to cultural inheritance. However, the study did observe that anomalies in beluga whale migration patterns were correlated with anomalous ice years.

### Dispersal and gene flow

The increased statistical power of the expanded data set that we used allowed a more quantitative assessment of sex-biased dispersal than earlier studies. While both sexes tend to be philopatric, we found that when dispersal does occur it is predominantly by adult males. Male-biased dispersal is typical in mammals and has been interpreted to be a strategy to reduce competition for mates and avoid inbreeding [8, 9, 84]. Large population and group sizes in Pacific beluga whales and opportunities to interbreed with other populations at certain times of year (see below) may largely reduce the need for male belugas to disperse. There is growing evidence for seasonal age and sex segregation in beluga whales and differences in habitat use between males and females [16, 22–25, 81]. In light of the present study’s findings it appears that both sexes may remain philopatric to the same migratory network but may not always travel in association.

While multiple factors confound comparisons of heterogeneity at markers with different modes of inheritance we were able to attribute the much lower nDNA differentiation compared to mtDNA differentiation among the subpopulations within the BCB region to greater male-mediated gene flow (see S1 Supporting information). We also, however, rejected panmixia on a common wintering ground. This differs from an earlier microsatellite study in this region by Brown Gladden et al. [27] based on a much smaller dataset (i.e., less locations, samples, years and loci). It also differs from recent findings on beluga whales in Canada and the Russian Far East. Turgeon et al. [75] found no convincing evidence of nDNA differentiation among demographically discrete summering assemblages in the Hudson Bay region concluding this likely reflected near random mating on a common breeding area. Similarly, Meschersky et al. [39] found no evidence of nDNA differentiation among two of the three demographically distinct summering aggregations in the Sea of Okhotsk (also see mtDNA findings in Table 3A this study) and concluded they share a common gene pool.

We found that genetic distance was more strongly related to distances between wintering rather than summer grounds suggesting gene flow and dispersal most likely occurred on or near the wintering grounds. Winter and early spring is the peak period of breeding [22] and the time of year when these discrete subpopulations are in closest proximity [31] (Fig 2). For example, while patterns of mtDNA variation in Norton Sound were consistent with a basin-wide pattern of female-mediated philopatry, patterns of nDNA differentiation and the outcomes of the model-based cluster analysis suggested extensive gene flow between Norton Sound and whales in the eastern Beaufort Sea and in the Bristol Bay subpopulations (Fig 3). While the summering grounds of these two subpopulations are far from Norton Sound (1,000km and 2,000km, respectively) a recent telemetry study revealed that the wintering range of the Norton Sound subpopulation may overlap to some degree with that of the eastern Beaufort Sea and Bristol Bay whales [31]. Similarly, low nDNA differentiation and clustering might suggest the same for the Anadyr Bay subpopulation and beluga whales in both the eastern Beaufort and the Sea of Okhotsk. However, low sample size from the latter precludes a well-supported inference at this time.

### Seasonal migration

The assignment tests and mixed stock analysis of migrating whales revealed that while both the wintering and summering areas of the eastern Chukchi Sea and eastern Beaufort Sea subpopulations may overlap somewhat [15, 16, 26, 31], the timing of spring migration differs such that the whales hunted at coastal sites in Chukotka, the Bering Strait (i.e., Diomede), and northwest Alaska (i.e., Point Hope) in the spring and off Alaska’s Beaufort Sea coast in summer were predominantly from the eastern Beaufort Sea population (Fig 5). This agrees with earlier genetic investigations [32] and recent telemetry studies [31] where the spring migration of eastern Beaufort whales occurs earlier and through denser sea ice than eastern Chukchi Sea belugas. The discovery that a few individual whales at some of these spring locations had higher likelihoods of eastern Chukchi Sea ancestry or of mixed-ancestry, however, indicates that the Bering Strait region is also an area of mixing in spring. This is also evident in recent movement data where Citta et al. [31] observed that for tagged eastern Beaufort Sea whales to migrate north in spring through the Bering Strait earlier than the eastern Chukchi belugas they had to pass through the latter’s primary wintering area.

### Temporal anomalies

The pattern of annual return to one traditional summering area in the BCB region, Kotzebue Sound, stands out from all others. The number of whales returning to Kotzebue Sound in summer dropped precipitously in the early-1980s [20]. This was followed by decades of inconsistent returns [85]. Earlier genetic studies found evidence that the pre-decline whales were likely a demographically distinct subpopulation and that more recent occurrences of belugas in the Sound in the 1990s and 2000s most likely involved whales from the Beaufort Sea [33, 83]. These studies, however, did not include all potential source subpopulations in the BCB region and so the origins of Kotzebue whales remained provisional. The current study included all major summer aggregations in the BCB region and confirmed that the pre-decline summer assemblage in Kotzebue Sound was likely a demographically discrete subpopulation and that beluga whales entering the Sound post-decline in the 1990s and 2000s (at least for those years where we had large sample sizes) were different from those we originally sampled and were most likely comprised of whales primarily from the eastern Beaufort Sea sub population.

### Evolution, ecology and management

The macro-geographic patterns of genetic differentiation in beluga whales across the North Pacific suggest divergence of the evolutionary trajectories of regional populations on millennial timescales. Regional adaptation of beluga populations is likely over such time frames while rescue of small and/or declining populations like that in Cook Inlet by whales from other regions is probably remote. The stability of heterogeneity among spatial strata on decadal time scales within regions and the evidence for generational-scale kinship within strata may indicate the kind of cultural stability in beluga whale populations that is necessary for gene-culture coevolution [4] where distinct cultures drive the evolution of neutral and functional genes over much faster time scales.

The study revealed that all spatially discrete summering aggregations within regions are demographically independent subpopulations and as such should be defined as separate stocks under management objectives to maintain healthy, functioning populations across the species range [86]. The greatly increased dataset over previous studies now means that five of the six summering aggregations within the BCB region can be more definitively defined as the following stocks: Bristol Bay, Eastern Bering Sea (Norton Sound), eastern Chukchi Sea (Kasegaluk Lagoon), eastern Beaufort Sea (Mackenzie-Amundsen), and Gulf of Anadyr (Anadyr Bay) (Table 1). We found that the sixth traditional summering area, Kotzebue Sound, was most likely a distinct subpopulation before declines in the 1980s but requires further investigation regarding its current stock composition.

Until now, limited knowledge on levels of interbreeding combined with a paucity of information on movements and distribution in late fall, winter and early spring shaped perceptions of beluga whale populations, and thus management units, across much of the Arctic that are dominated by summer distributions [20, 37, 38, 87]. We define stocks, for example, by their primary coastal summering location.

This study’s findings, in concert with new satellite telemetry data [18, 31], force us to alter this perception. The demographically distinct summering aggregations within the BCB region, for example, in most cases: (1) do not appear to interbreed extensively, (2) return to discrete wintering areas, and (3) disperse and interbreed over limited distances. Thus, beluga whales in the Bering, Chukchi and Beaufort Seas comprise a series of populations or subpopulations with discrete, traditional migratory circuits and destinations that overlap to varying degrees at certain times of year. As well as challenging current concepts on connectivity between subpopulations within regions, these findings will also alter our approach to estimating effective population sizes (N_e_) and how to model risk, recovery and population viability. Furthermore, when the migratory circuits of these subpopulations are considered in light of the heterogeneity of Pacific Arctic and subarctic marine ecosystems [88] the individual subpopulations likely also have quite distinct ecologies and perhaps other unique aspects to their behavior and population biology. It follows then that different migratory populations are likely exposed to different threats.

Finally, the study confirms the critical importance of the Northern Bering Sea and Bering Strait region to beluga whales. This relatively small area (Fig 2) is home to tens of thousands of beluga whales from multiple subpopulations, some among the largest in the world, for example [89, 90], for much of the year. This region is also important for other marine mammal species (Citta et al. in review.) and is currently undergoing dramatic environmental and ecosystem change [88, 91]. Minor shifts in this region’s environment and ecosystem, including sea-ice and productivity, could have major impacts on beluga whale dispersal, breeding behavior, population status and structure across much of their Pacific range.

## Supporting information

S1 Supporting information This is a file that contains supporting information text. (DOCX)

S1 Fig Median Joining network of beluga whale mtDNA haplotypes across their North Pacific Ocean. (TIF)

S2 Fig Summary plot of model-based cluster analyses of population structure in Pacific beluga whales with N_max_ = 100 (A) compared to whales with N = all (B). (TIF)

S3 Fig Estimation of the likely number of population clusters, K, using the rate of change in the log probability of the data, the ΔK statistic of Evanno. (TIF)

S4 Fig Temporal cluster analysis of nDNA data from beluga whales in the Pacific using STRUCTURE. For those summering concentrations where we had genotypes from more than one decade individuals from each summering location were assigned to the same population cluster. Each of 973 individuals is represented by a vertical line with estimated membership, Q, in each cluster denoted by different colors. The analysis was based on eight microsatellite loci, used prior sample group information (LOCPRIOR), and yielded similar results for both the admixture and no admixture (shown) models of ancestry. For each geographic stratum the decade 1988–1997 is denoted by a grey bar across the top of the figure, and the decade 1998–2007 is denoted by a white bar.
(TIF)

S1 Table Estimates of genetic diversity and probabilities of identity for eight microsatellite loci in beluga whales. Values are given for the major beluga whale concentration areas in the western Nearctic, and for the combined spring migration (i.e., Point Hope) and summering concentration (I.e. Mackenzie-Amundsen) areas for the Beaufort Sea.
(XLSX)

S2 Table Summary of results from STRUCTURE analysis of microsatellite data from beluga whales in the North Pacific. The likelihoods of K given the data is reported for 28 separate analyses, each with unique parameter settings. Analyses were run both with and without admixture, with and without sampling location included as prior information, and they contained sample sets of varying size randomly sampled using R (n = 15, 30, 50, 100, and all) that included individuals genotyped at a range of loci (n ≥ 6 loci, n ≥ 7 loci, n = all 8 loci). For each parameter set we used a burn-in period of 50,000 iterations followed by 2x105 iterations to collect data. Multiple runs (n = 5) were conducted for each value of K (n = 8) to ensure convergence for a total of 1,120 separate runs. The most likely K for each analysis is shaded and details on the clustering is provided in the comments. Analyses where K = 6 was well supported are shaded in blue.
(XLSX)

S3 Table Correlation between geographic distance and genetic distance among seasonal ranges of beluga whales in the Bering, Chukchi and Beaufort Seas for both mtDNA and microsatellites. Mantel tests, based on 10,000 permutations were conducted in Arlequin 3.5.
(XLSX)

S4 Table Point estimates (mean and median) and Bayesian posterior probability bounds (95%) of population composition estimates, p, of beluga whales migrating past four locations in the Bering, Chukchi and Beaufort Seas in spring and early summer using the stock-mixture program BAYES. Baseline samples are from likely migration destinations, the eastern Beaufort Sea and the eastern Chukchi Sea. Multiple chains (1,800–12,000 reps) were run and shrink factors did not exceed 1.01.
(XLSX)

S5 Table Genetic estimates of recent immigration into beluga whale populations. Means (± 95% confidence intervals) of the posterior distribution of the proportion of individuals that are migrants (m) based on the analysis of multi-locus genotypic data in BayesAss. To insure convergence 10 separate runs, each with 20 x 106 iterations, the first 1 x 106 iterations of which were burn-in, were conducted. Receiving populations are in rows, delta = 0.15 and n denotes sample size. Similar results were found when the Beaufort Sea was represented by whales sampled only at the Mackenzie Delta-Amundsen Gulf.
(XLSX)

S6 Table Identification of candidate migrants and individuals with likely migrant ancestry using Bayesian and maximum-likelihood assignment tests. Bayesian inference was based on multilocus genotype probabilities using the model-based clustering method, STRUCTURE (MCMCs involved a burn-in period of 30,000 rep.s followed by 1 X 106 rep.s.) and the BayesAss program (MCMC runs with 20 X 106 rep.s, the first 1 X 106 discarded as burn-in). Likelihoods were estimated in WHICHRUN and are reported as log10 ratios of the likelihood that an individual's haplo-genotype was more likely in a population other than where it was sampled assuming random assortment of microsatellite alleles and that observed mtDNA haplotypic frequencies and microsatellite allele frequencies represent population frequencies. Relative likelihoods were estimated with the Assignment Calculator program in DOH where P represents the proportion of 1,000 new individuals randomly drawn from each population which had equal or smaller likelihood values. Only results for the sampled population (i.e., baseline) and the population the STRUCTURE and WHICHRUN analysis assigned the individual to are presented. Only individuals that had low probabilities of non-immigrant ancestry (at either ν = 0.05 or 0.1) and/or had high mtDNA-nDNA LOD scores (≥1) are reported.
(XLSX)

S7 Table Temporal patterns of genetic differentiation (Fst) within mitochondrial DNA (A) and within microsatellite loci (B) in Pacific beluga whales. **The temporal strata comprised the following four 10-year sample windows: 1980s = 1978–1987); 1990s = 1988–1997); 2000s = 1998–2007); and 2010s (≥ 2008). P-values from homogeneity tests, based on 50,000 permutations, are represented by the following shading patterns: dark grey: p≤0.01, light grey: 0.01<p≤0.05, unshaded: p>0.05. Only strata with a sample size of n≥10 are reported. The boxes bordered in blue highlight pairwise comparisons across decades within geographic strata.** 50,175 permutations.
(XLSX)

S1 Appendix A more detailed summary of sample sizes of beluga whales from the same fifteen geographic strata in the North Pacific Ocean shown in Table 1. Here, the samples are broken out by decadal time periods. The last two columns summarize the total sample sizes for the primary sampling period from 1988–2010 and includes samples from Meschersky et al. (2013).
(DOCX)
