# Supplementary material for: Migratory culture, population structure and stock identity in North Pacific beluga whales (Delphinapterus leucas)
Source: PLoS One. 2018 Mar 22;13(3):e0194201. doi: 10.1371/journal.pone.0194201 (PMC5863979; doi:10.1371/journal.pone.0194201)
Supplement: S1 Supporting information — (DOCX) [file pone.0194201.s001.docx]

**Supporting Information**

**Migratory culture, population structure and stock identity of North Pacific beluga whales (*Delphinapterus leucas*)**

Greg O’Corry-Crowe, Robert Suydam, Lori Quakenbush, Brooke Potgieter, Lois Harwood, Dennis Litovka, Tatiana Ferrer, John Citta, Vladimir Burkanov, Kathy Frost, Barbara Mahoney

Supporting Information figures are provided as separate files. Also, Supporting Information tables are provided in a separate Excel file.

**Materials and Methods**

Dispersal patterns over ecological time scales were investigated using the Bayesian approach of Wilson and Rannala [1] in the program BayesAss 1.3. Recent immigration rates (with 95% confidence intervals) were estimated from posterior probabilities generated from the genotypic data using MCMC techniques. Multiple (n=10) long (20 x 10^6^ iterations) runs with large sampling frequencies (2000) and different initial seeds and long (1 x 10^6^) burn-in periods were run to ensure convergence. This method allows genotypic frequencies of baseline populations to deviate from H-W equilibrium proportions and accommodates for missing data.

To assess levels of relatedness across sample year and generation within an area, and to compare proportions of first and second-order relationships (based on nDNA) within and between years we first used the program coancestry [2] to calculate seven different moment and likelihood estimators of relatedness, r, from the multilocus genotype data, including those of Queller & Goodnight [3] and Wang [4]. Bootstrap analysis (100,000 reps) over loci was used to obtain confidence in intrapair estimators and intergroup comparisons of average relatedness. We then used ml-relate [5] to calculate ML estimates of relationship as well as relatedness among pairs of individuals in order to test whether the proportion of distant/unrelated pairs to three categories of close relationship: parent offspring (r=0.5), full-sib (r=0.375) and half-sib or equivalent (r=0.25), differed among years compared to within year.

**Results**

MtDNA and nDNA diversity

Estimates of genetic diversity and probabilities of identity for eight microsatellite loci are provided in S1 Table.

A total of 252 tests were run in Genpop 4.1, comprising 8 loci tested against each other, equaling 28 tests, across nine geographic strata. Using α=0.05, we ran a Markov Chain analysis with the following parameters: dememorization of 10,000, 20 batches with the number of iterations per batch equaling 5,000. Of the 252 tests, 19 had P<0.05 comprising 7.54% of tests. Following Bonferroni correction for multiple comparisons (m=9) of the same hypothesis for each locus pair, only 7 tests had p< α/m comprising 2.78 percent of tests.

Median joining network of mtDNA haplotypes

A median joining network of unique mtDNA haplotypes found among beluga whales sampled across their North Pacific range (S1 Fig). We chose a subset of samples (n=1,383) and haplotypes (n=36) that span all summering concentration areas and other strata in the Pacific Ocean and include all the common lineages to illustrate the primary aspects of Pacific beluga whale mtDNA phylogeography: (1) a series of star-like phylogenies and (2) limited spatial distribution of some lineages. The inclusion of all samples and lineages does not alter the outcome substantively (not shown).

The network was created using NETWORK 4.6 software (Fluxus Technology Ltd.) and was characterized by a series of star-like phylogenies with several rarer haplotypes radiating from a more common central haplotype. Haplotype labels are indicated beside each node. Node size represents overall haplotype frequencies and the proportion of each haplotype found in each of 5 major geographic strata in the north Pacific is presented by a different color; Cook Inlet (green), Bristol Bay (red), Norton Sound (orange), Kasegaluk Lagoon (yellow) and the Mackenzie Delta-Amundsen Gulf (black).

Genetic differentiation and model-based cluster analysis

In a hierarchical AMOVA of population structure in Arlequin for frequency-based F-statistics, none of the proportion of the total mtDNA variance observed among populations (i.e., *F*_ST_ = 0.3059) was due to differentiation among regional groupings (-0.04%, *F*_CT_=-0.004), all was due to variance among populations within regions (30.63%, *F*_SC_= 0.3061). For distance-based statistics, the proportion of the total mtDNA variance observed among populations (i.e., Φ_ST_ = 0.305) that was due to differentiation among regional groupings was higher (1.88%, Φ_CT_=-0.019) but still the majority was due to variance among populations within regions (30.63%, Φ_SC_= 0.292).

The hierarchical AMOVA found a greater proportion of the total variance observed among populations (*F*_ST_) was due to differentiation among regional groupings (*F*_CT_) with the microsatellite data compared to mtDNA. This was true for both frequency (*F*_ST_ = 0.0558, *F*_CT_ 0.0437, *F*_SC_ = 0.0127) and distance-based statistics (*R*_ST_ = 0.098, *R*_CT_ 0.086, *R*_SC_ = 0.014).

We conducted a total of 28 distinct model-based cluster analyses of population structure in Pacific beluga whales using Structure 2.3.4, each with a unique set of modelling assumptions. Each analysis comprised 5 independent runs for each of 8 values of *K* for a total of 1,120 separate runs. We provide summaries of the findings in Table S2 below.

We used Clumpak [6] to generate summary plots of the model-based cluster analysis of population structure in Pacific beluga whales that we conducted using Structure, and to implement two methods for choosing *K*. Optimal cluster alignments across multiple independent runs (n=5) for different values of *K* were genreated for each Structure analysis. Here we present the major modes for just *K*=4, 5 and 6 out of the total range tested (i.e., *K*=1-8) (S2A and S2B Figs) for two of those analysis: (A) the analysis using prior sample group information, no admixture, all samples scored at ≥6 loci and a maximum sample size of n=100, and (B) the similar analysis using all available samples. Both analyses revealed *K*=5 clusters as the most likely (see panel 2 in both A and B). However, in a number of analyses *K*=6 was the most or second-most likely resulting in the separation of Anadyr into a discrete cluster (see panel 3 in each case).

We implemented two methods for the preferred choice of *K* using Clumpak; that by Evanno et al., [7] and that by Pritchard et al. [8] and Hubisz et al. [9], and present our findings (see S3 Fig) for the analysis summarized in S2A Fig above. The ΔK statistic of Evanno et al. [7] found that the greatest rate of change in the log probability of the data (excluding prior population information) was for K=2 (see S3 Fig) while Structure’s Probability of K for the analysis that included prior population information was highest for K=5.

Mantel tests revealed positive correlations between genetic differentiation and geographic distance within the BCB region. Stronger correlations with genetic heterogeneity were found among wintering areas compared to summering areas and for mtDNA compared to nDNA (S3 Table).

Seasonal migration

The stock-mixture method, Bayes, estimated population proportions and thus likelihoods of migrating groups being of mixed origin, and assigned individual migrants probabilistically to baseline populations. S4 Table summarizes the point estimates, mean, median and Bayesian posterior probability bounds of population composition estimates of four migrating groups in the BCB region.

Dispersal - rates

Genetic estimates of recent dispersal rates using BayesAss indicated negligible immigration (m≤0.004) into Cook Inlet from other populations (S5 Table). Estimates (and confidence intervals) were more varied among summering concentrations within the Bering, Chukchi and Beaufort Seas, with a substantial proportion of individuals identified as immigrants or of immigrant ancestry. We found, however, that the scale and direction of migration rates among the four BCB summering areas was influenced greatly by the starting assumptions including the number of strata included in the analysis and the size of the delta value (0.15-0.30). The uncertainty within BCB suggests that, unlike the comparisons between Cook Inlet and BCB, there was not enough information in the data to estimate rates of dispersal within BCB over such a short time frame (a few generations) using this method.

Dispersal – Individual assignments

Genetic estimates of recent dispersal rates using BayesAss indicated negligible immigration (m≤0.004) into Cook Inlet from other populations (S5 Table). By contrast, uncertainty over estimates among summering concentrations within BCB suggests that, unlike the comparisons between Cook Inlet and BCB, there was not enough information in the data to estimate rates of dispersal within BCB over such a short time frame (a few generations) using this method.

Patterns of recent dispersal were also characterized using individual-assignment methods. Using four likelihood and Bayesian inference methods very few individuals were identified as possible migrants or of migrant ancestry (S6 Table). Only 24 out of 661 individuals (i.e., 3.63%) with complete mtDNA-microsatellite profiles were assigned by Whichrun to a population other than the one they were sampled in at LOD scores ≥1 (S6 Table). Just four of these whales were cross-assigned at LOD ≥2: three from the eastern Chukchi Sea, one from Cook Inlet. When relative likelihoods of the genotypes within each population were taken into account using Assignment Calculator, most individuals could not be excluded from their nominal population. When individual assignments were viewed in relation to the distribution of likelihoods of 1,000 generated individuals within each baseline population, only eight individuals possessed genotypes estimated to be relatively rare (*P*<0.1) in their nominal population (S6 Table). Using population information to test for migrants and setting the prior probability that an individual was an immigrant (or had a recent immigrant ancestor) to *v* = 0.05, Structure did not identify a single individual whose probability that it originated exclusively in the population where it was sampled was < 0.5 (not shown). At *v* = 0.1 only two animals, an adult male from the eastern Chukchi Sea (#4418) and adult female from Cook Inlet (#24855), had moderate probabilities of being a migrant or having migrant ancestry from Norton Sound (*Pr* = 0.123) and Bristol Bay (*Pr* = 0.205), respectively (S6 Table). Finally, individual assignments based on posterior probability distributions of immigrant ancestries in BayesAss, yielded extremely low assignment proportions of many Norton Sound and Beaufort Sea individuals to their nominal populations, including individuals cross-assigned by the other methods (Table S6), further cautioning against the ability of BayesAss analysis to measure contemporary dispersal patterns within the BCB region. BayesAss did reveal that the two Cook Inlet whales identified by whichrun as being of likely migrant origin had low probabilities of having migrant ancestry (0.145 - 0.308) from the BCB populations (S6 Table).

Temporal and kinship analysis

Temporal comparisons on decadal scales revealed almost no change in the geographic patterns of mtDNA and nDNA variation for almost all geographic strata over ecological time frames (S7 Table and S4 Fig).

**Discussion**

Median joining network of mtDNA haplotypes

The network of mtDNA haplotypes was characterized by a series of star-like phylogenies with several rarer haplotypes radiating from more common central haplotypes, a pattern widely interpreted as indicative of ancient population expansions [10, 11]. Furthermore, an earlier study of the demographic history of Atlantic beluga whales that included two Pacific populations found that the distribution of pairwise differences among mtDNA haplotypes in each case were consistent with an evolutionary history of population expansion [12].

Cluster analysis

The cluster analysis revealed a number of surprising results that require further exploration. Specifically, on the one hand the Okhotsk Sea stratum in some analysis clustered with strata in the BCB region and with Cook Inlet in the Gulf of Alaska Region. By contrast, Bristol Bay and Kasegaluk Lagoon *within* the BCB region often emerged early on (i.e., at low values of K) as separate population clusters in the sequence of cluster analyses. There may be a number of factors at play here, including sample size, drift, *K*, the assumptions of the cluster analysis, and demographic history. The clustering is probably influenced greatly by how similar individuals within certain sample sets are to each other and how less similar others in other sample sets are. This can be influenced by a populations’ demographic history, population size, and by sampling. Looking at K=4 in S2B Fig the resident population in Cook Inlet emerges early on due presumably to ancient divergence, drift, and/or similar genotypes. Bristol Bay also emerges early too and possibly for the same reasons suggesting it may have had a somewhat discrete glacial refugium. Surprisingly, Kasegaluk also emerges at this stage. By contrast the Okhotsk sample does not emerge until K=6! Why? Well, we know from the relatedness analysis that we have likely sampled a lot of close relatives in the Kasegaluk lagoon stratum. Conversely, we have small sample sets from the other strata that likely include few close relatives. So Kasegaluk pops out first because: (1) it may be quite distinct from all other strata, possibly having a different glacial refugium and/or unique demographic history, (2) it has large sample sizes and thus better defines it’s underlying allele frequencies, (3) it has a substantial number of animals with very similar genotypes helping the analysis define it as a separate cluster early on. The issue with the Okhotsk Sea may be the reverse: (1) we can’t exclude possible ancestral connections to other BCB strata, (2) it has small sample sizes, and (3) it has whales with quite different genotypes making it difficult to identify a distinct Okhotsk cluster.

Dispersal and gene flow

While lower differentiation at nuclear compared to mtDNA loci may reflect male-biased dispersal or gene flow on common breeding sites [13, 14], and has been widely interpreted to indicate just that [15, 16, 17], such inferences are complicated by differences in the rates of genetic drift between nuclear and cytoplasmic markers, and by mutation rate and thus the particular suite of nuclear markers chosen. Fortunately, in our study the inclusion of the geographically isolated and phylogeographically distinct Cook Inlet population, where both male and female dispersal rates are likely negligible on contemporary time frames, helps distinguish among the effects of drift, mutation rate and sex-biased dispersal. Estimates of microsatellite heterogeneity for those pair-wise comparisons involving Cook Inlet were 4 to 7 times lower than corresponding estimates of mtDNA heterogeneity. By contrast, mean microsatellite differentiation among BCB strata were 19 to 25 times lower than corresponding mtDNA estimators. Considering the evolutionary-level distinctness of the Cook Inlet population, these ratios suggest that the lower nDNA compared to mtDNA differentiation observed between Cook Inlet and other sub-populations is primarily due to differences in the rate of genetic drift and the choice of loci, not male-biased dispersal or interbreeding. All else being equal (i.e. drift, mutation, mating system) the much lower nDNA relative to mtDNA differentiation observed among the Bering, Chukchi and Beaufort Sea summering groups likely reflects greater gene flow.

**References**

1. Wilson GA, Rannala B. Bayesian inference of recent migration rates using multilocus genotypes. Genetics. 2003; 163: 1177–1191.
2. Wang J. COANCESTRY: a program for simulating, estimating and analyzing relatedness and inbreeding coefficients. Mol Ecol Resour*.* 2011; 11: 141-145.
3. Queller DC, Goodnight KF. Estimating relatedness using molecular markers. Evolution. 1989; 43: 258–275.
4. Wang J. An estimator for pairwise relatedness using molecular markers. Genetics. 2002; 160: 1203–1215.
5. Kalinowski ST, Wagner AP, Taper ML. ml-relate: a computer program for maximum likelihood estimation of relatedness and relationship. Mol Ecol Notes. 2006; 6: 576-579.
6. Kopelman N.M., Mayzel J., Jakobsson M., Rosenberg N.A., Mayrose I. Clumpak: a program for indetifying clustering modes and packaging population structure inferences across K. Mol Ecol Resour. 2015; 15: 1179-1191.
7. Evanno G, Regnaut S Goudet J. Detecting the number of clusters of individuals using the software structure: a simulation study. Mol Ecol. 2005; 15: 2611-2620.
8. Pritchard JK, Stephens P, Donnelly P. Inference of population structure using multilocus genotype data. Genetics. 2000; 155: 945–959.
9. Hubisz MJ, Falush D, Stephens M, Pritchard JK. Inferring weak population structure with the assistance of sample group information. Mol Ecol Resour. 2009; 9: 1322–1332.
10. Lavery S, Moritz C, Fielder DR. Indo-Pacific population structure and evolutionary history of the coconut crab *Birgus latro*. Mol Ecol. 1996; 5: 557-570.
11. O’ Corry-Crowe GM, Suydam RS, Rosenberg A, Frost KJ, Dizon AE. Phylogeography, population structure and dispersal patterns of the beluga whale *Delphinapterus leucas* in the western Neartic revealed by mitochondrial DNA. Mol Ecol. 1997; 6: 955-970.
12. O’Corry-Crowe GM, Lydersen C, Heide-Jørgensen MP, Hansen L, Mukhametov LM, Dove O, et al. Population genetic structure and evolutionary history of North Atlantic beluga whales (*Delphinapterus leucas*) from West Greenland, Svalbard and the White Sea. Polar Biol. 2010; 33: 1179-1194.
13. Melnick DJ, Hoelzer GA. Differences in male and female macaque dispersal lead to contrasting distributions of nuclear and mitochondrial DNA variation. Int J Primat. 1992; 13: 379–393.
14. Prugnolle F, de Meeus Y. Inferring sex-biased dispersal from population genetic tools: a review. Heredity. 2002; 88: 161-165.
15. Karl SA, Bowen BW, Avise JC. Global population genetic structure and male-mediated gene flow in the green turtle (*Chelonia mydas*): RFLP analyses of anonymous nuclear loci. Genetics. 1992; 131: 163–173.
16. Brown Gladden JG, Ferguson MM, Freisen MK, Clayton JW. Population structure of North American beluga whales (*Delphinapterus* *leucas*) based on nuclear DNA microsatellite variation and contrasted with population structure revealed by mitochondrial DNA. *Mol Ecol*. 1999; 8: 347–363.
17. Escorza-Trevino S, Dizon AE. Phylogeography, intraspecific structure and sex-biased dispersal of Dall’s porpoise, *Phocoenoides dalli*, revealed by mitochondrial and microsatellite DNA analysis. Mol Ecol. 2000; 9: 1049-1060.
