# Supplementary figures and images for: Migratory culture, population structure and stock identity in North Pacific beluga whales (Delphinapterus leucas)
Source: PLoS One. 2018 Mar 22;13(3):e0194201. doi: 10.1371/journal.pone.0194201 (PMC5863979; doi:10.1371/journal.pone.0194201)

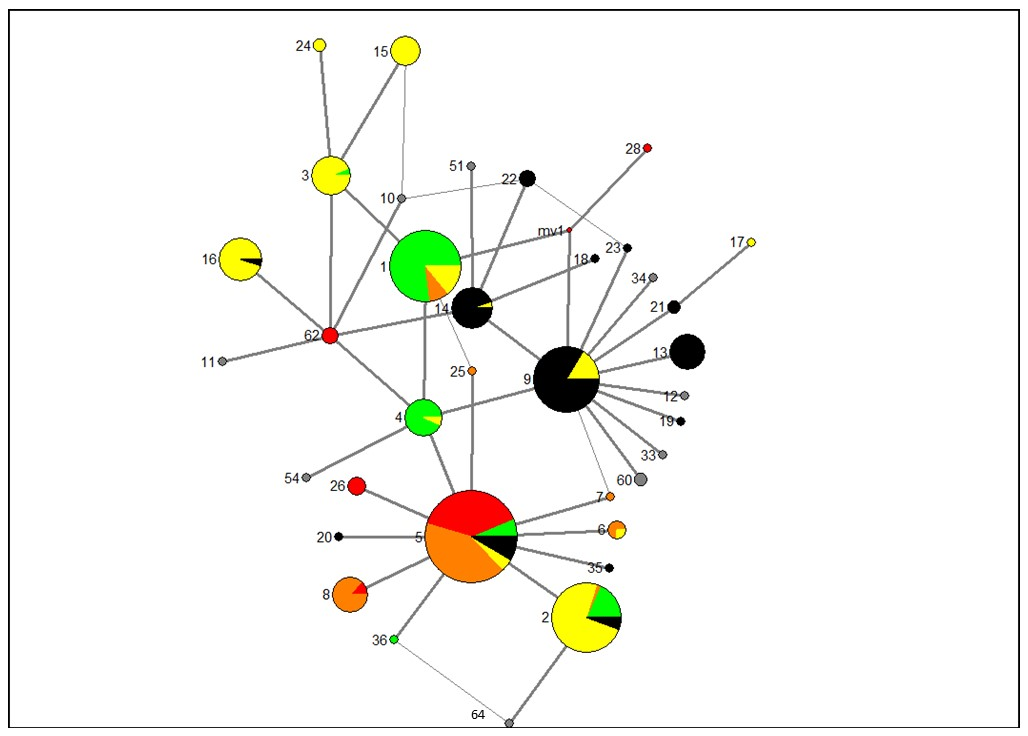

Supplement: S1 Fig — (TIF) [file pone.0194201.s002.tif]

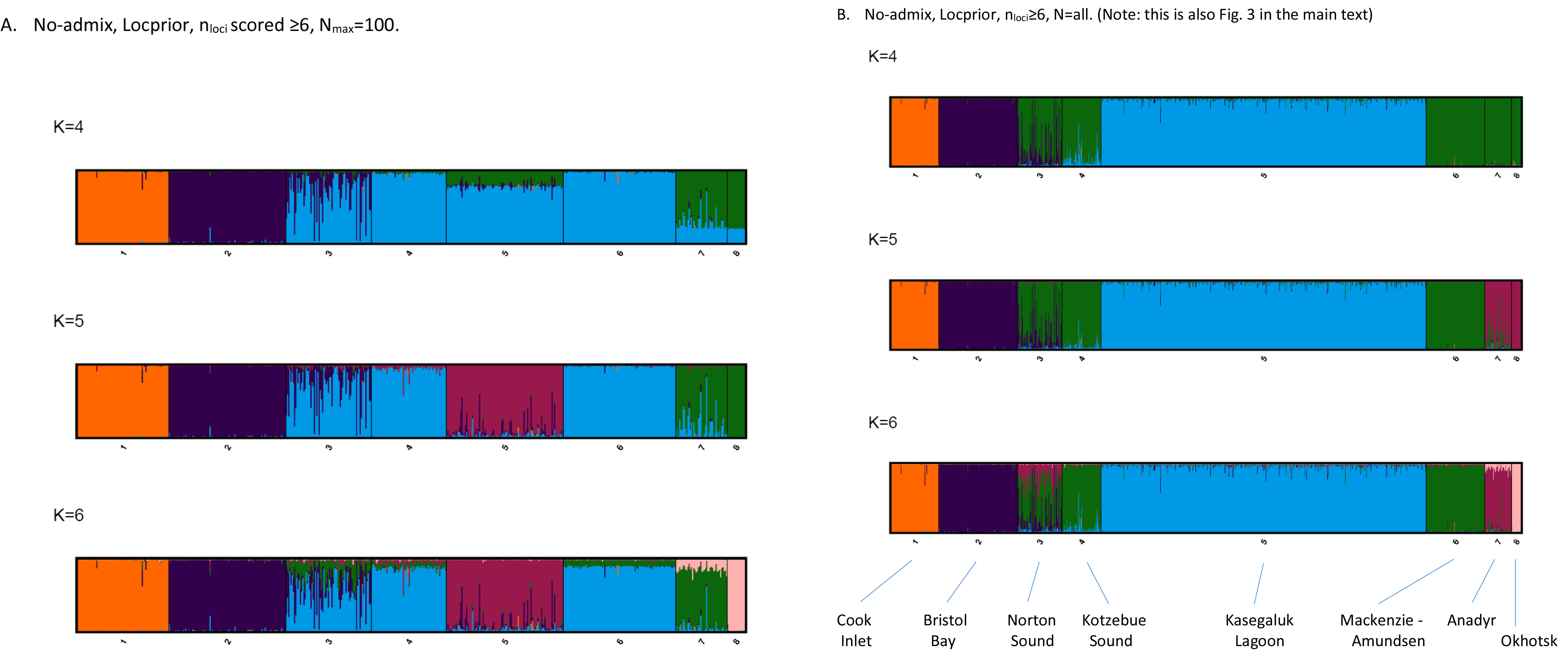

Supplement: S2 Fig — (TIF) [file pone.0194201.s003.tif]

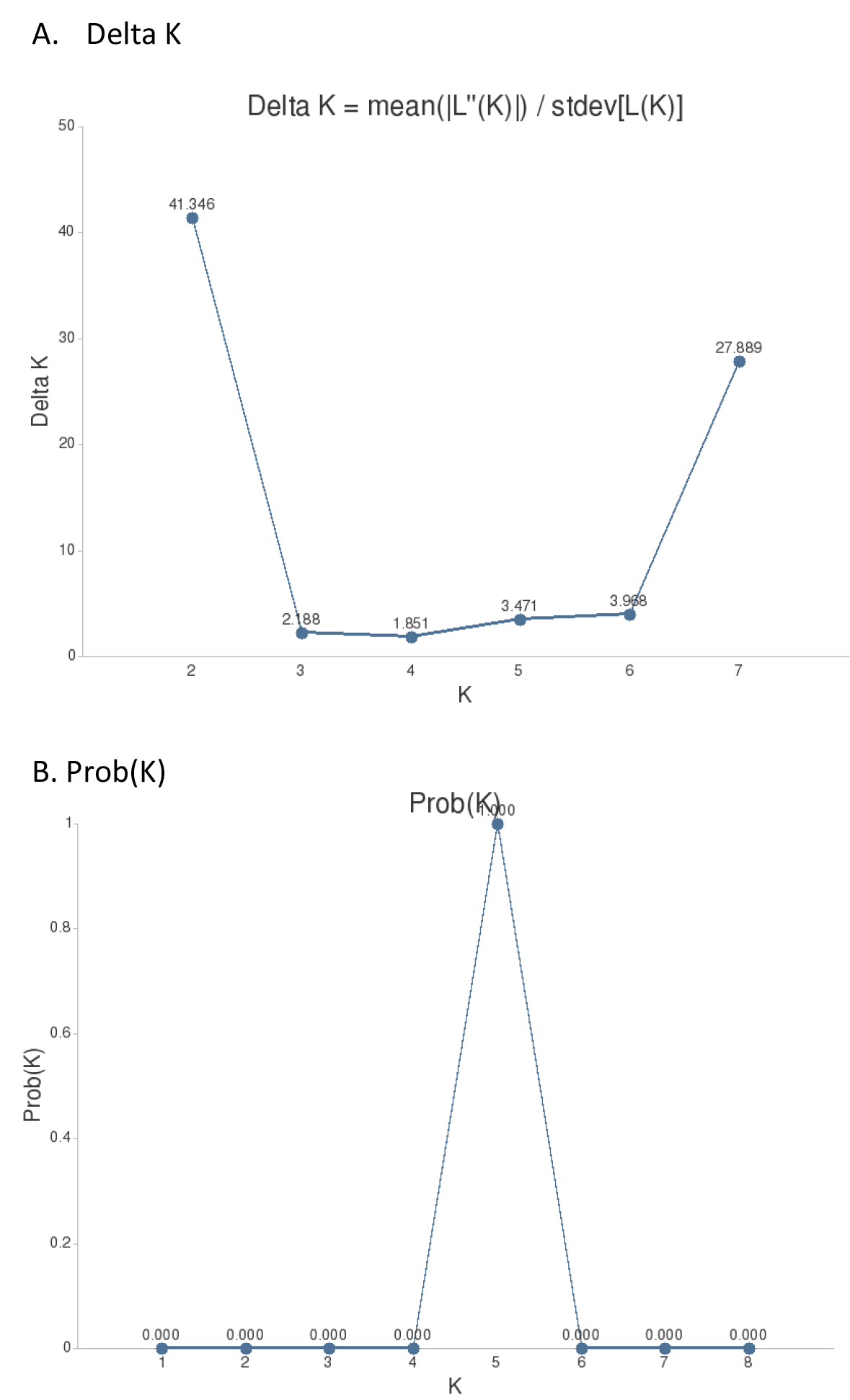

Supplement: S3 Fig — (TIF) [file pone.0194201.s004.tif]

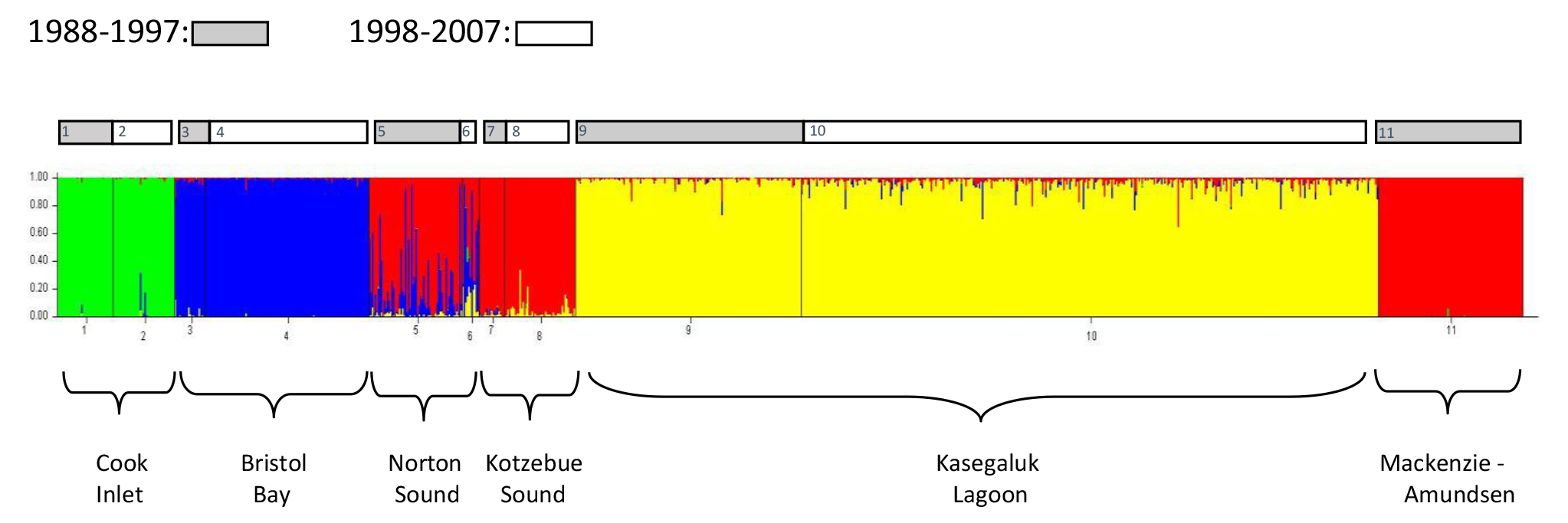

Supplement: S4 Fig — For those summering concentrations where we had genotypes from more than one decade individuals from each summering location were assigned to the same population cluster. Each of 973 individuals is represented by a vertical line with estimated membership, Q, in each cluster denoted by different colors. The analysis was based on eight microsatellite loci, used prior sample group information (LOCPRIOR), and yielded similar results for both the admixture and no admixture (shown) models of ancestry. For each geographic stratum the decade 1988–1997 is denoted by a grey bar across the top of the figure, and the decade 1998–2007 is denoted by a white bar. (TIF) [file pone.0194201.s005.tif]
