# Supplementary material for: Migratory culture, population structure and stock identity in North Pacific beluga whales (Delphinapterus leucas)
Source: PLoS One. 2018 Mar 22;13(3):e0194201. doi: 10.1371/journal.pone.0194201 (PMC5863979; doi:10.1371/journal.pone.0194201)
Supplement: S1 Appendix — Here, the samples are broken out by decadal time periods. The last two columns summarize the total sample sizes for the primary sampling period from 1988–2010 and includes samples from Meschersky et al. (2013). (DOCX) [file pone.0194201.s013.docx]

Appendix 1. A more detailed summary of sample sizes of beluga whales from the same fifteen geographic strata in the north Pacific Ocean shown in Table 1. Here the samples are broken out by decadal time periods. The last two columns summarize the total sample sizes for the primary sampling period from 1988-2010 and includes samples from Meschersky et al. (2013).

|  |  |  |  |  |  |  |  |  |  |  |  |  |  | MtDNA |  | primary sampling | |
| --- | --- | --- | --- | --- | --- | --- | --- | --- | --- | --- | --- | --- | --- | --- | --- | --- | --- |
| **Region** | **Geographic Strata** | **Mitochondrial DNA** | | | | |  | **Microsatellites** | | | | |  | (Meschersky |  | period | |
|  |  | 1978-1987 | 1988-1997 | 1998-2007 | 2008-2009 | **total** |  | 1978-1987 | 1988-1997 | 1998-2007 | 2008-2009 | **total** |  | et al. 2013) |  | **mtDNA** | **Micro.s** |
| Gulf of Alaska | Cook Inlet |  | 48 | 85 |  | **133** |  |  | 37 | 41 |  | **78** |  |  |  | *133* | *78* |
|  | Yakutat Bay |  |  | 8 |  | **8** |  |  |  | 8 |  | **8** |  |  |  | *8* | *8* |
|  |  |  |  |  |  |  |  |  |  |  |  |  |  |  |  |  |  |
| Bering-Chukchi-Beaufort | Bristol Bay |  | 26 | 114 |  | **140** |  |  | 20 | 109 |  | **129** |  |  |  | *140* | *129* |
|  | Norton Sound |  | 104 | 87 |  | **191** |  |  | 62 | 11 |  | **73** |  |  |  | *191* | *73* |
|  | Kotzebue Sound | 39 | 24 | 56 |  | **119** |  |  | 17 | 47 |  | **64** |  |  |  | *80* | *64* |
|  | Kasegaluk Lagoon | 38 | 158 | 383 |  | **579** |  |  | 150 | 383 |  | **533** |  |  |  | *541* | *533* |
|  | Mackenzie-Amundsen |  | 101 |  |  | **101** |  |  | 96 |  |  | **96** |  |  |  | *101* | *96* |
|  | Anadyr |  |  | 15 | 31 | **46** |  |  |  | 13 | 31 | **44** |  | 37 |  | *83* | *44* |
|  |  |  |  |  |  |  |  |  |  |  |  |  |  |  |  |  |  |
| Okhotsk | western Kamchatka |  |  |  | 4 | **4** |  |  |  |  | 4 | **4** |  | 14 |  | *18* | *4* |
|  | Sakhalinskiy Bay |  |  | 12 |  | **12** |  |  |  | 1 |  | **1** |  | 106 |  | *118* | *1* |
|  | Shantar - Udskaya Bay |  | 10 |  |  | **10** |  |  | 10 |  |  | **10** |  | 46 |  | *56* | *10* |
|  |  |  |  |  |  |  |  |  |  |  |  |  |  |  |  |  |  |
| BCB Migration | eastern Chukotka |  |  | 8 | 2 | **10** |  |  |  | 9 |  | **9** |  |  |  | *10* | *9* |
|  | Little Diomede Island |  |  | 11 |  | **11** |  |  |  | 10 |  | **10** |  |  |  | *11* | *10* |
|  | Point Hope | 29 | 24 | 2 |  | **55** |  | 13 | 22 |  |  | **35** |  |  |  | *26* | *22* |
|  | Barrow-Kaktovik |  | 10 | 6 |  | **16** |  |  | 10 | 3 |  | **13** |  |  |  | *16* | *13* |
|  |  |  |  |  |  |  |  |  |  |  |  |  |  |  |  |  |  |
|  | cow-calf pairs |  | 9 |  |  | **9** |  |  | 9 |  |  | **9** |  |  |  | *9* | *9* |
|  |  |  |  |  |  |  |  |  |  |  |  |  |  |  |  |  |  |
|  | Total: | 106 | 514 | 787 | 37 | **1444** |  | 13 | 433 | 635 | 35 | **1116** |  |  |  | 1541 | 1103 |
